# Regulation of ER stress-induced apoptotic and inflammatory responses via YAP/TAZ-mediated control of the TRAIL-R2/DR5 signaling pathway

**DOI:** 10.1038/s41420-025-02335-w

**Published:** 2025-02-04

**Authors:** Y. El Yousfi, F. J. Fernández-Farrán, F. J. Oliver, A. López-Rivas, R. Yerbes

**Affiliations:** 1 Centro Andaluz de Biología Molecular y Medicina Regenerativa-CABIMER, CSIC-Universidad de Sevilla-Universidad Pablo de Olavide, Seville, Spain; 2 Instituto de Parasitología y Biomedicina López Neyra, CSIC, Centro de Investigación Biomédica en Red de Cáncer CIBERONC, Granada, Spain; 3 Present Address: Medical Physiology and Biophysics Department, Universidad de Sevilla and Instituto de Biomedicina de Sevilla (IBiS) (Hospital Universitario Virgen del Rocío/CSIC/Universidad de Sevilla), Seville, Spain

**Keywords:** Cancer microenvironment, Apoptosis

## Abstract

In tumors, cancer cells are frequently exposed to adverse environmental conditions that result in endoplasmic reticulum (ER) stress. Mechanical signals emerging from extracellular matrix (ECM) rigidity and cell shape regulate the activity of transcriptional co-activators Yes-associated protein (YAP) and its paralog Transcriptional Coactivator with PDZ-binding motif (TAZ). However, the role of ECM rigidity and YAP/TAZ in tumor cell fate decisions under ER stress remains relatively unexplored. Our results suggest that the YAP/TAZ system plays an important role in the control of ER stress-induced cell death by mechanical signaling arising from ECM stiffness in tumor cells. Mechanistically, YAP/TAZ regulates apoptosis induced by ER stress in tumor cells by controlling the activation of the TRAIL-R2/DR5-mediated extrinsic apoptotic pathway through a dual mechanism. On the one hand, the YAP/TAZ system prevents intracellular TRAIL-R2/DR5 clustering in tumor cells. On the other hand, it inhibits cFLIP down-regulation in tumor cells experiencing ER stress. In addition, YAP/TAZ controls the expression of pro-inflammatory interleukin-8 (IL-8/CXCL8) in tumor cells undergoing ER stress by a TRAIL-R2/DR5/caspase-8-dependent mechanism. Although other mechanisms may also be involved in controlling cell death and inflammation in tumor cells facing environmental stress, our results support a model in which regulation of the subcellular localization and activity of the YAP/TAZ transcriptional co-activators could contribute to the microenvironmental control of cell fate decisions in tumor cells undergoing ER stress.

## Introduction

Overall, tumor microenvironment differs significantly from normal tissue [1]. In solid tumors, the extracellular matrix (ECM) of the tumor microenvironment plays a critical role in the adaptive response to overcome the various types of inherent stresses and the resistance of tumor cells to therapy [2–4]. Moreover, increasing evidences indicate that the mechanical properties of the ECM are important determinants of tumor cell fate when facing the different stressors of the tumor microenvironment [1, 5]. Among the multiple physical parameters, matrix rigidity can especially have an impact on intracellular signaling pathways, influencing cancer progression and the tumor response to therapy [5, 6].

In response to environmental and physiologic stress conditions that increase the load of unfolded proteins in the ER [3], protein sensors located in the luminal face of the ER membrane activate the unfolded protein response (UPR) [7]. Activation of the UPR leads to a reduction in the entry of proteins into the ER, triggers protein degradation pathways, and enhances the folding capacity of the ER to allow adaptive and repair processes that re-establish homeostasis [8]. However, above a certain threshold, unresolved ER stress results in the activation of an apoptotic cell death process [9, 10].

In tumor cells experiencing chronic ER stress, cell death by apoptosis could be preceded by a decrease in anti-apoptotic proteins levels and/or up-regulation of the expression of pro-apoptotic proteins of the Bcl-2 family [11, 12]. In addition, a TNF-related apoptosis-inducing ligand (TRAIL)-independent intracellular activation of TRAIL receptor 2 (TRAIL-R2/DR5)-mediated extrinsic apoptotic pathway following ER stress has been demonstrated [13–15]. More recently, it was reported that misfolded proteins directly bind to and activate TRAIL-R2/DR5 in the ER–Golgi intermediate compartment to induce caspase-8 activation and apoptosis [16].

YAP (yes-associated protein 1) and TAZ (transcriptional coactivator with PDZ-binding motif) are highly related transcriptional regulators that play a critical role as downstream transducers of the Hippo pathway to control organ size [17] and tumor development [18] through binding to and activation of transcription factors of the TEAD family [19]. Some evidence has indicated that Hippo pathway-independent regulation of YAP/TAZ by mechanotransduction [20, 21] may be involved in controlling the resistance of tumor cells to different therapeutic strategies [22, 23]. However, the potential role of YAP/TAZ in mediating matrix stiffness-regulated responses to microenvironmental cues leading to ER stress remains to be established. Furthermore, although a role of YAP in the control of the apoptotic response to ER stress has been reported [24], the downstream effector mechanism remains largely unknown.

In this study, we found that ECM rigidity controls ER stress-induced activation of TRAIL-R2/DR5-mediated apoptotic signaling in tumor cells by a YAP/TAZ-regulated mechanism. Our data also reveal that the YAP/TAZ-TEAD transcriptional module may play a role both in the control of apoptosis and the pro-inflammatory response activated in tumor cells experiencing ER stress. Collectively, our results suggest a key function of the YAP/TAZ transcriptional co-activators on tumor cell fate in the adverse conditions of the tumor microenvironment through the control of ER stress-induced TRAIL-R2/DR5-mediated signaling pathways.

## Results

### Matrix rigidity modulates ER stress-induced cell death in tumor cells by controlling the activation of the TRAIL-R2/DR5 signaling pathway

**Fig. 1:**
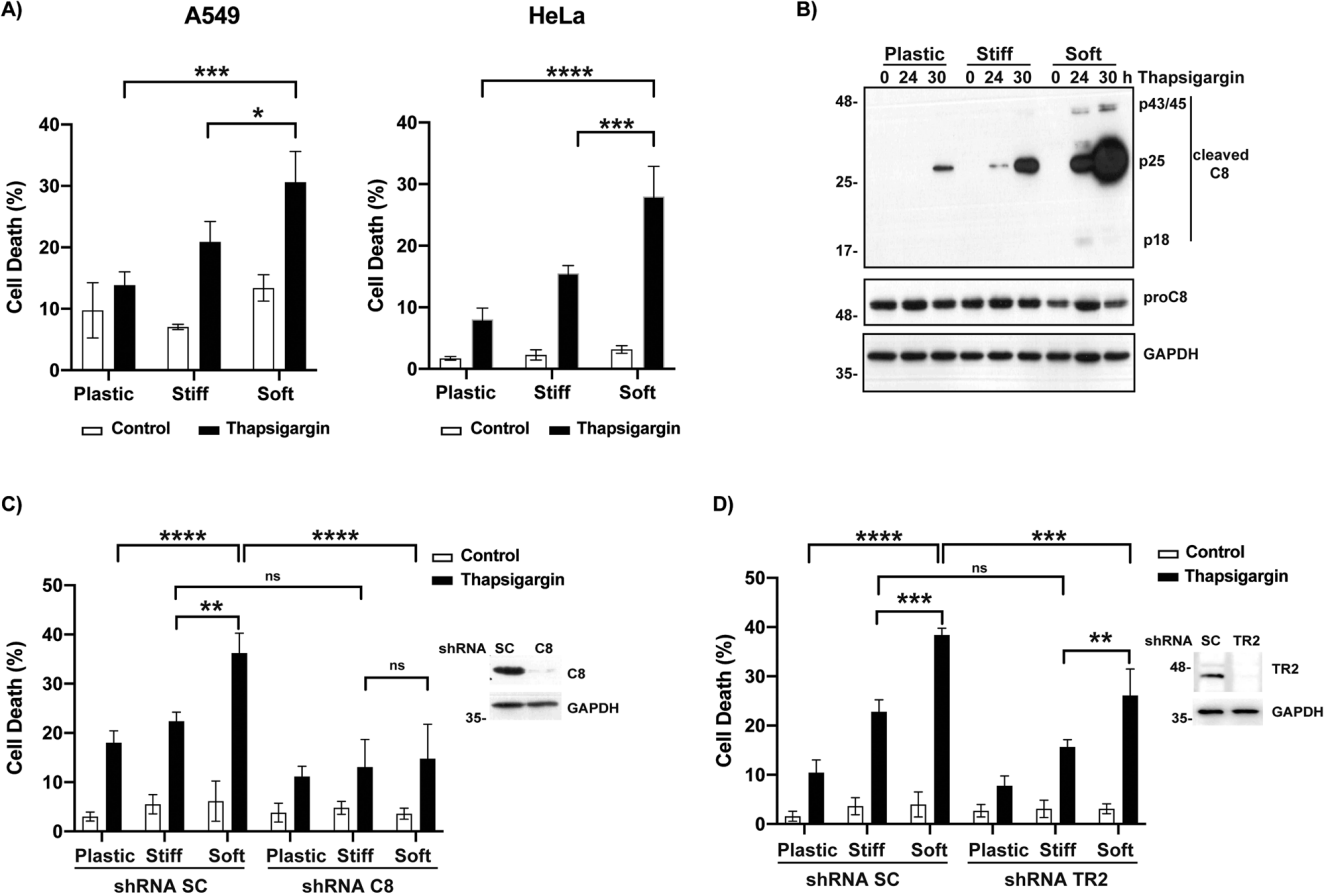
ECM stiffness modulates ER stress-induced cell death. **A** Cells were plated on plastic or collagen-coated polyacrylamide gels with different rigidity as described in Material and Methods and treated with thapsigargin 200 nM during 48 h. Cell death was analyzed by quantification of subG1 population (A549) or PI uptake (HeLa). **B** A549 cells were treated as in (**A**) and activation of caspase-8 (C8) was assessed by western blotting. shScrambled (SC) or shcaspase-8 (C8) A549 cells (**C**) and shScrambled (SC) or shTRAIL-R2 (TR2) A549 cells (**D**) were treated as in (**A**) and cell death was assessed by quantification of subG1 population. Knockdown of C8 and TR2 was assessed by western blotting. Data represent mean ± SD of at least three independent experiments. *P < 0.05; **P < 0.01; ***P < 0.001; ****P < 0.0001, Two-way ANOVA with Tukey´s multicomparisons test.

Chronic ER stress-induced upon unfolded or misfolded protein build-up in the ER activates the PERK/ATF4/CHOP/TRAIL-R2/DR5-dependent and TRAIL-independent intracellular assembly of the death-inducing signaling complex (DISC) leading to a caspase-8-mediated apoptotic pathway [13–15]. To assess whether the ECM rigidity could play a role in the response of tumor cells to sustained ER stress we first evaluated the apoptotic response to the ER stress inducers thapsigargin and tunicamycin in tumor cells growing on gel substrates mimicking soft and rigid ECM. Cells growing on plastic culture plates were also included as a control substrate of very high rigidity (2 GPa). As shown in Fig. 1A and Supplementary Fig. 1A, tumor cells growing on stiff ECM or plastic were significantly more resistant to ER stress-induced apoptosis than cells cultured on soft ECM. Consistent with these findings, caspase-8 activation as assessed by determining the proteolytic processing of pro-caspase-8 upon thapsigargin treatment, was clearly enhanced in A549 cells cultured in soft ECM (Fig. 1B). Given the increased activation of caspase-8 upon ER stress in A549 cells growing under conditions of low rigidity, we investigated the role of caspase-8 and the TRAIL-R2/DR5-mediated apoptotic pathway in the mechanism underlying the enhanced sensitivity to ER stress of tumor cells growing in a soft ECM. As shown in Fig. 1C caspase-8 knockdown by RNA interference prior to thapsigargin treatment markedly inhibited apoptosis and abrogated the differential sensitivity to sustained ER stress in A549 cells cultured in the various substrates. Furthermore, silencing TRAIL-R2/DR5 expression significantly inhibited thapsigargin-induced apoptosis in A549 cells growing in a low-rigidity ECM (Fig. 1D).

### Role of YAP/TAZ transcriptional coactivators in the control of ER stress-induced apoptosis by matrix stiffness

**Fig. 2:**
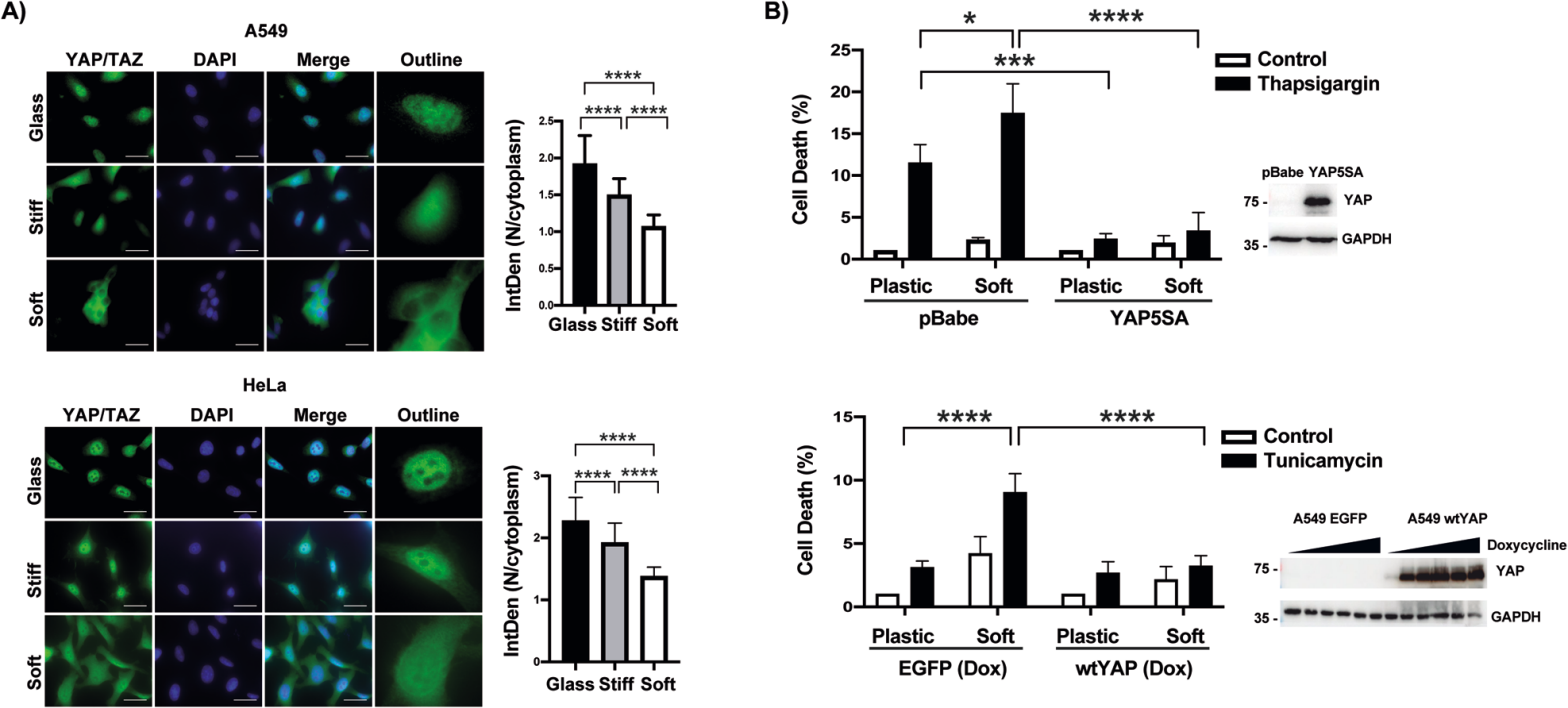
Role of YAP/TAZ in the control of ER stress-induced cell death by ECM stiffness. **A** Representative images of immunofluorescence from A549 and HeLa cells plated on collagen-coated polyacrylamide gels with different rigidity and showing YAP/TAZ localization (scale bars 20 µm). Graphs show nuclear/cytosolic YAP/TAZ ratio quantification. Data are derived from three independent experiments where at least 300 cells were scored. Error bars represent mean ± SD. **B** pBabe or YAP5SA A549 cells (upper panel) and EGFP or wtYAP A549 cells treated with 1 µg/ml of doxycycline (lower panel) were seeded on plates of different stiffness and incubated in medium with or without thapsigargin (200 nM) for 48 h or tunicamycin (1 µg/ml) for 72 h, respectively. Cell death was assessed by quantification of subG1 population. Data are normalized to control. YAP expression was analyzed by western blotting. Data show the mean ± SD of five independent experiments. *P < 0.05; **P < 0.01; ***P < 0.001; ****P < 0.0001, Two-way ANOVA with Tukey´s multicomparisons test.

Data from different studies have indicated that YAP/TAZ transcriptional coactivators play an important role as nuclear messengers of mechanical signals regulated by ECM rigidity [20, 21]. To get further insight into the mechanism of the regulation of sensitivity of tumor cells to ER stress by matrix stiffness we initially assessed by immunofluorescence the subcellular localization of endogenous YAP/TAZ in A549 and HeLa cells growing either on acrylamide hydrogels of varying rigidity or plastic. As reported in other cell types [20, 21], nuclear YAP/TAZ levels were significantly higher in A549 and HeLa cells grown on stiff hydrogels or plastic as compared to those cells on a soft substrate (Fig. 2A). Mechanical cues control YAP/TAZ nuclear localization by Hippo dependent and independent mechanisms [20, 21, 25]. To further assess the importance of YAP/TAZ on the regulation of ER stress-induced apoptosis by matrix stiffness, we generated A549 cells expressing a constitutively active form of YAP (YAP5SA) lacking inhibitory LATS phosphorylation sites to prevent YAP inactivation by the Hippo pathway [19]. As shown in Fig. 2B, A549-YAP5SA cells were resistant to ER stress in a manner independent of substrate rigidity (Fig. 2B, upper panel). Furthermore, expression of an inducible form of wild-type YAP (wtYAP) significantly inhibited tunicamycin-induced apoptosis in cells grown on soft hydrogels (Fig. 2B, lower panel).

**Fig. 3:**
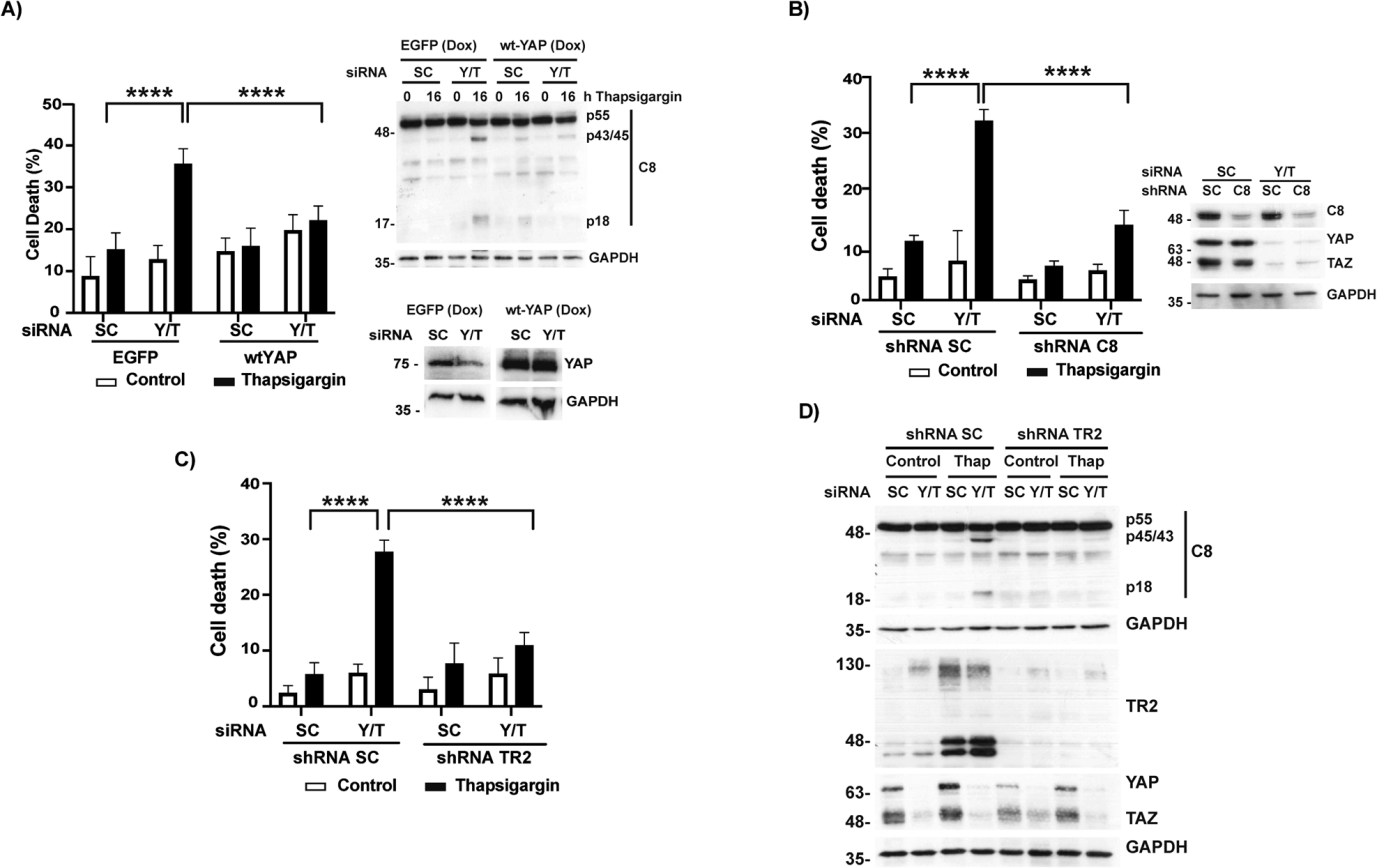
YAP/TAZ modulates TRAIL-R2/caspase-8 apoptotic signaling upon ER stress. **A** A549 EGFP or A549 wtYAP cells were transfected with siRNAs against both YAP and TAZ (Y/T) as described in “Material and Methods”. A scrambled (SC) RNA was also used as non-targeting control oligonucleotide. After 6 h, cells were treated with doxycycline 1 µg/ml. Thirty hours post-transfection, thapsigargin 200 nM was added during 24 h. Cell death was measured as quantification of subG1 population. Caspase-8 (C8) activation and YAP knockdown were analyzed by western blotting. shScrambled (SC) or shcaspase-8 (C8) A549 cells (**B**) and shScrambled (SC) or shTRAIL-R2 (TR2) A549 cells (**C**) were transfected with SC or Y/T siRNAs. After 30 h, cells were treated with thapsigargin 200 nM during 24 h. and cell death was assessed by quantification of subG1 population. Protein knockdown was verified by immunoblot analysis. **D** shSC or shTR2 A549 cells were treated as in (**C**) and C8 activation was assessed by western blotting. Data represent mean ± SD from three independent experiments. *P < 0.05; **P < 0.01; ***P < 0.001; ****P < 0.0001, Two-way ANOVA with Tukey´s multicomparisons test.

To further examine the impact of reduced nuclear levels of YAP/TAZ in the apoptotic response to ER stress, we silenced YAP/TAZ expression by siRNA in A549 cells growing on a high-rigidity plastic substrate prior to thapsigargin treatment. As shown in Fig. 3A and Supplementary Fig. 1B, C, knockdown of endogenous YAP/TAZ clearly sensitized different tumor cell lines to thapsigargin. Importantly, sensitization to ER stress by YAP/TAZ siRNAs was significantly inhibited in tumor cells expressing a siRNA-insensitive form of wild-type YAP (Fig. 3A and Supplementary Fig. 1D). In addition, YAP/TAZ silencing facilitated thapsigargin-induced activation of caspase-8 that was prevented in cells expressing siRNA-insensitive wild-type YAP (Fig. 3A). In agreement with the results showing a dependency on caspase-8 and TRAIL-R2/DR5 of the increased sensitivity to ER stress of cells grown on a soft ECM, sensitization of tumor cells to thapsigargin treatment upon YAP/TAZ knockdown was markedly inhibited in cells that had been silenced for the expression of either caspase-8 (Fig. 3B and Supplementary Fig. 2A) or TRAIL-R2/DR5 (Fig. 3C). The role of TRAIL-R2/DR5 in YAP/TAZ-mediated regulation of ER stress-induced apoptosis in A549 cells was further assessed by analyzing caspase-8 processing upon thapsigargin treatment. Strikingly, YAP/TAZ-depleted A549 cells in which TRAIL-R2/DR5 expression was stably silenced by shRNA showed a marked resistance to thapsigargin-induced caspase-8 activation (Fig. 3D).

Collectively, all these results suggest a key role of mechanically controlled YAP/TAZ transcriptional coactivators in the regulation of tumor cell response to ER stress by restraining the activation of the TRAIL-R2/DR5 signaling pathway.

### Activity of the YAP/TAZ-TEAD signaling module determines resistance to ER stress-induced apoptosis in tumor cells

**Fig. 4:**
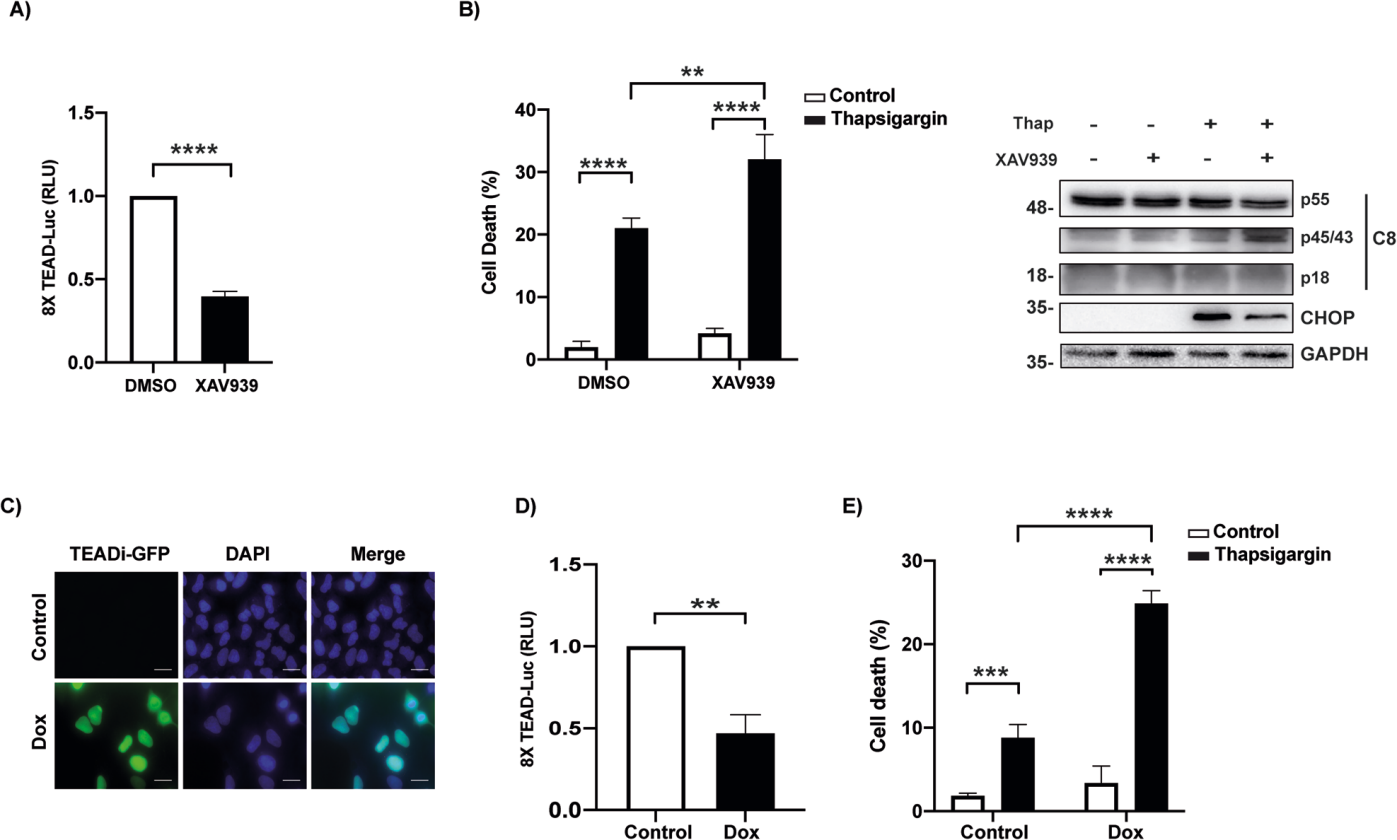
YAP/TAZ-TEAD signaling confers resistance to ER stress-induced apoptosis in tumor cells. **A** A549 cells were cultured in the presence or absence of XAV939 5 μM during 24 h and transcriptional activity of TEAD was measured by luciferase assay with 8XGTII–lux reporter. **B** A540 cells were treated as in (**A**) and then thapsigargin 200 nM was added during 24 h. Cell death was analyzed by quantification of subG1 population. CHOP expression and caspase-8 processing were assessed by western blotting. **C** Microscope images showing TEADi-GFP expression in A549 cells after doxycycline treatment (1 μg/ml, 24 h) (scale bars 20 µm). **D** TEAD transcriptional activity measured by luciferase assay in TEADi-GFP A549 cells cultured in the presence or absence of doxycycline 1 μg/ml 24 h. **E** A549 TEADi-GFP cells were treated with doxycycline during 24 h before treatment with thapsigargin for 24 h. Cell death was measured as quantification of subG1 population. Data show the mean ± SD of at least three independent experiments. *P < 0.05; **P < 0.01; ***P < 0.001; ****P < 0.0001, Unpaired *T*-test (**A**, **D**); Two-way ANOVA with Tukey´s multi comparisons test (**B**, **E**).

Once in the nucleus, YAP/TAZ interact with different transcription factors to induce the expression of genes involved in cell proliferation, cell differentiation, apoptosis, and stemness [26]. Among the various transcription factors that can be regulated by YAP/TAZ, members of the TEAD family are major players in the execution of the transcriptional program [27]. Regulation of YAP/TAZ-TEAD activity by matrix stiffness is an evolutionarily conserved mechanism, shared by multiple cell types [20]. On the other hand, angiomotin family proteins have been reported to translocate nuclear YAP into the cytoplasm [28]. Tankyrases target the angiomotin proteins to promote their degradation [29]. Furthermore, the tankyrase inhibitor XAV939 has been shown to reduce YAP/TEAD activity in different tumor cell lines by translocating YAP from the nucleus into the cytoplasm [29]. To gain a deeper understanding of the mechanism controlling sensitivity of tumor cells to ER stress-induced apoptosis by YAP/TAZ, we first assessed the effect of XAV939 on YAP/TAZ-TEAD activity. Results shown in Fig. 4A indicate that XAV939 clearly inhibited TEAD luciferase reporter activity in A549 tumor cells. Importantly, treatment of A549 cells with the tankyrase inhibitor significantly sensitized these tumor cells to thapsigargin-induced caspase-8 processing and apoptosis (Fig. 4B).

A genetically encoded tetracycline-inducible inhibitor of the interaction of YAP and TAZ with TEAD transcription factors (TEADi) has recently been used to investigate YAP/TAZ-TEAD-mediated transcriptional effects [30]. To further assess the role of TEAD transcription factors in the control of ER stress-induced apoptosis, we generated A549 cells expressing tetracycline-inducible GFP-tagged TEADi by lentiviral transduction (Fig. 4C) as described in “Materials and Methods” section. TEADi expression upon addition of doxycycline inhibited TEAD luciferase reporter activity (Fig. 4D). Importantly, a marked sensitization to ER stress-induced apoptosis was observed in A549 cells lentivirally transduced with TEADi plasmid, following doxycycline treatment (Fig. 4E).

### Intracellular clustering of TRAIL-R2/DR5 and down-regulation of cFLIP levels upon inhibition of YAP/TAZ-TEAD signaling

**Fig. 5:**
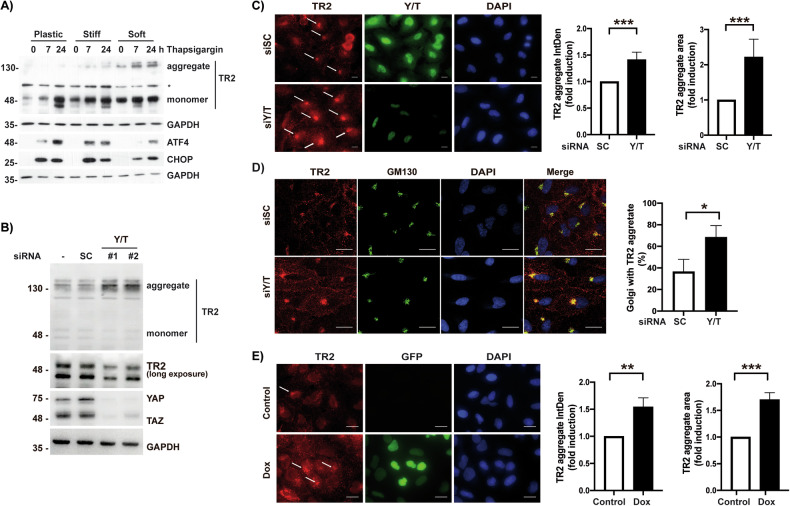
Limiting YAP/TAZ-TEAD signaling promotes intracellular TRAIL-R2/DR5 clustering. **A** A549 cells were plated on plastic or collagen-coated polyacrylamide gels with different rigidity as described in Material and Methods and treated with thapsigargin (200 nM) for the indicated times. Protein expression was analyzed by western blotting. **B** A549 cells were transfected either with a scrambled (SC) oligonucleotide or with two different siRNAs against both YAP/TAZ (Y/T) and protein expression was assessed by western blotting 48 h after transfection. **C** Representative images of TRAIL-R2/DR5 (TR2) and YAP/TAZ (Y/T) immunofluorescence from A549 cells transfected with Y/T siRNAs or SC siRNA oligonucleotide. White arrows indicate TRAIL-R2/DR5 aggregates (scale bars 20 µm). Graph shows TRAIL-R2/DR5 aggregates integrated density and area quantification. **D** Representative confocal microscopy images of Golgi localized TRAIL-R2/DR5 from A549 cells transfected either with SC siRNA or Y/T siRNAs (scale bars 20 µm). Graph shows co-localization of Golgi marker GM130 with TRAIL-R2/DR5 aggregates. **E** Immunofluorescence images of TRAIL-R2/DR5 aggregates from A549 TEADi cells treated with doxycycline during 24 h. Graph shows TRAIL-R2/DR5 aggregates integrated density and area quantification.

Collectively, our results point to a role of the YAP/TAZ-TEAD module in the control of TRAIL-R2/caspase-8 signaling and the cell death response in tumor cells undergoing chronic ER stress. ER stress inducers are known to activate the extrinsic apoptotic pathway through the PERK-mediated induction of the CHOP transcription factor, leading to the upregulation of TRAILR2/DR5 expression and TRAIL-independent intracellular clustering and activation of TRAIL-R2/DR5 in tumor cells [13–15]. In a different context, it was reported that YAP/TAZ-TEAD signaling is required for the expression of genes involved in cytoskeleton remodeling and protein trafficking from the Golgi to the plasma membrane [31]. However, the impact of YAP/TAZ-TEAD inhibition on the subcellular localization of TRAIL-R2/DR5 remains unexplored. To address this issue, we first determined the effect of matrix stiffness on the activity of the PERK-ATF4-CHOP arm of the UPR, the canonical proapoptotic pathway activated in cells undergoing chronic ER stress, in A549 cells treated with thapsigargin. Although there were no clear differences in PERK branch signaling upon thapsigargin treatment between cells grown on substrates of different rigidity, high molecular weight TRAIL-R2/DR5 oligomers were detected mainly in cells growing on low rigidity hydrogels that were further increased upon ER stress (Fig. 5A). Both TRAIL-dependent and independent clustering of TRAIL-R2/DR5 are known to induce an apoptotic response [16, 32, 33]. To investigate the role of YAP/TAZ in the control of the UPR and TRAIL-R2/DR5 oligomerization, A549 cells were depleted of YAP/TAZ with two different siRNAs targeting YAP/TAZ prior to thapsigargin treatment. Despite causing a clear sensitization to ER stress-induced cell death (Supplementary figure 2B), YAP/TAZ knockdown did not affect the activation of the UPR as measured by assessing eIF2-alpha phosphorylation and expression of ATF4, CHOP, and GRP78 proteins (Supplementary Fig. 2C). Likewise, silencing YAP/TAZ expression had no impact on TRAIL-R2/DR5 mRNA expression (Supplementary Fig. 2D). However, as previously observed in cells grown on soft hydrogels (Fig. 5A), silencing YAP/TAZ expression in cells grown on plastic markedly induced TRAIL-R2/DR5 oligomerization (Fig. 5B). Additional evidence of intracellular TRAIL-R2/DR5 clustering upon YAP/TAZ knockdown was obtained by immunofluorescence analysis. Silencing YAP/TAZ expression by RNA interference significantly induced intracellular TRAIL-R2/DR5 clustering (Fig. 5C and Supplementary Fig. 3A) at the Golgi apparatus (Fig. 5D). Furthermore, results shown in Supplementary Fig. 3B reveal that YAP/TAZ knockdown induces a significant increase on TRAIL-R2/DR5 clustering upon thapsigargin treatment. Likewise, silencing YAP/TAZ expression clearly sensitized these cells to tunicamycin-induced TRAIL-R2/DR5 oligomerization and caspase-8 processing (Supplementary Fig. 3C). Importantly, TRAIL-R2/DR5 clustering was also observed in TEADi cells upon doxycycline treatment (Fig. 5E), suggesting a role of the YAP/TAZ-TEAD module in TRAIL-R2/DR5 trafficking.

**Fig. 6:**
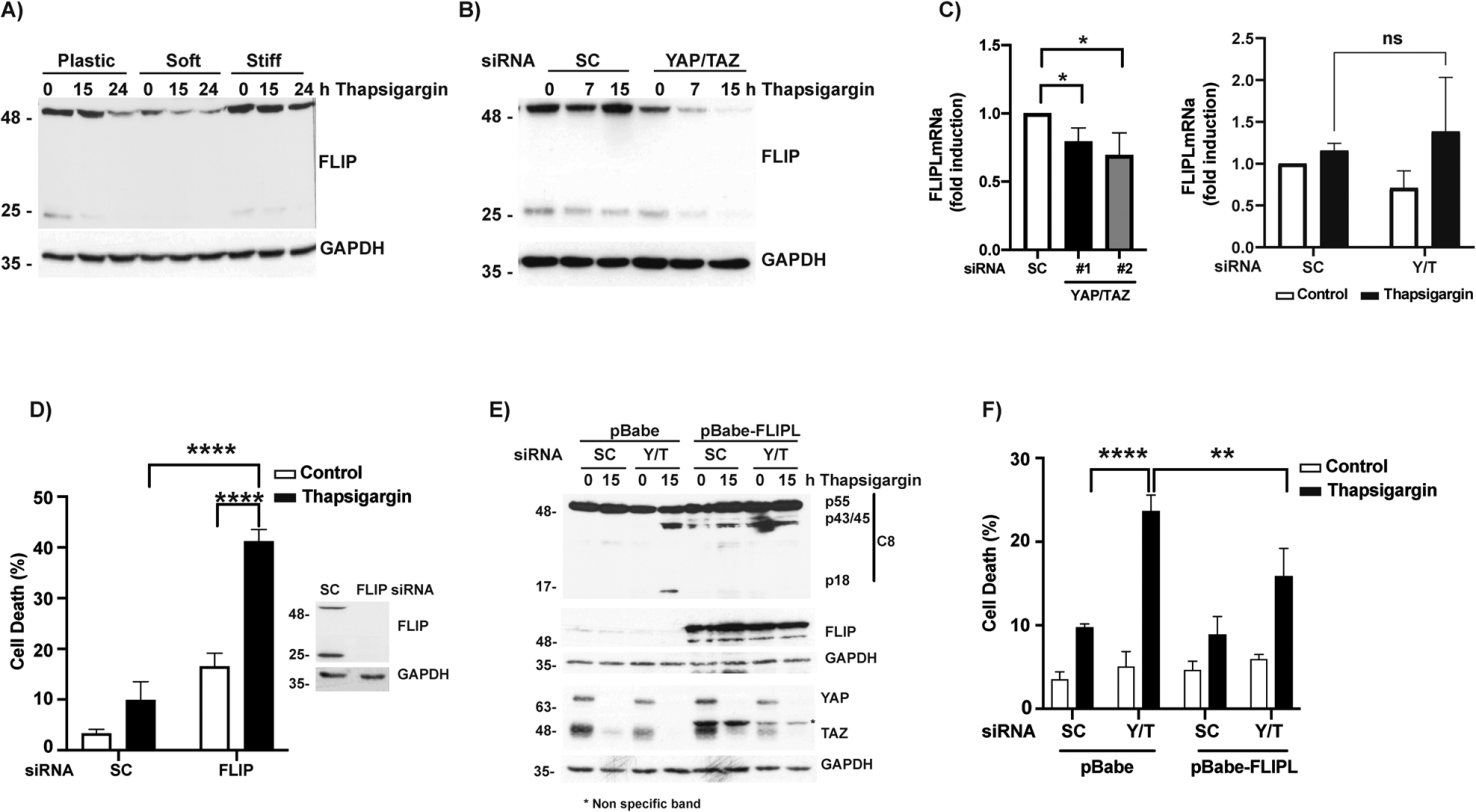
Control of cFLIP expression levels by ECM stiffness and YAP/TAZ in A549 cells. **A** A549 cells were plated on plastic or collagen-coated polyacrylamide gels with different rigidity as described in Material and Methods and treated with thapsigargin 200 nM during the indicated time points. Protein expression was analyzed by western blotting. **B** A549 cells were transfected with a scrambled oligonucleotide (SC) or siRNA YAP/TAZ (Y/T). Thirty hours post-transfection, cells were treated with thapsigargin and protein expression was analyzed by western blotting. **C** A549 cells were transfected with two different siRNA against both YAP and TAZ. 48 h post-transfection, cFLIP mRNA levels were assessed by qPCR (left panel). In right panel, A549 cells were transfected with siRNA targeting YAP/TAZ, and 30 h after transfection cells were treated overnight with thapsigargin, and cFLIP mRNA levels were assessed by qPCR. Data show the mean ± SD of from three independent experiments (**D**) A549 cells were transfected with siRNA oligonucleotides targeting both cFLIP isoforms. After 6 h cells were treated with thapsigargin for 24 h. Cell death was analyzed by subG1 quantification. Data show the mean ± SD of at least three independent experiments. **E** A549 pBabe or A549pBabe-FLIPL were transfected with siRNA SC or siRNA Y/T. After 30 h, cells were treated with thapsigargin 200 nM during 24 h. Caspase-8 (C8) activation was analyzed by western blotting. **F** Cells were treated as in (**E**) and cell death was analyzed by quantification of subG1 population. Data represent mean ± SD from three independent experiments. *P < 0.05; **P < 0.01; ***P < 0.001; ****P < 0.0001. Unpaired Ttest (**C**), Two-way ANOVA with Tukey´s multicomparisons test (**D**, **F**).

The antiapoptotic proteins cFLIP_L_ and cFLIP_S_ are key regulators of apoptosis triggering upon TRAIL receptors activation by TRAIL [34–36] and ER stress [37]. To get further insight into the mechanism of matrix stiffness-mediated control of ER stress-induced apoptosis, we examined cFLIP levels in cells grown on substrates of different rigidity. As shown in Fig. 6A, levels of both cFLIP_L_ and cFLIP_s_ proteins were significantly reduced in cells grown on a soft ECM as compared to cells grown on a stiff hydrogel or plastic. We next explored the effect of YAP/TAZ silencing on cFLIP expression in A549 cells grown on a plastic substrate. As shown in Fig. 6B, C knockdown of YAP/TAZ led to a significant decrease in cFLIP protein and mRNA levels. Expression of cFLIP protein was further reduced upon thapsigargin treatment in cells grown on a soft ECM (Fig. 6A) and YAP/TAZ knockdown cells (Fig. 6B). Unlike protein levels, analysis of cFLIP mRNA levels in YAP/TAZ depleted cells did not show a decrease after treatment with thapsigargin (Fig. 6C). Next, we assessed the relevance of cFLIP down-regulation observed in cells grown on soft hydrogels or depleted of YAP/TAZ by siRNA in the sensitization to ER stress-induced apoptosis. To this end, we silenced cFLIP expression in A549 cells prior to treatment with thapsigargin. As shown in Fig. 6D, silencing of both cFLIP isoforms markedly sensitized these cancer cells to thapsigargin-induced apoptosis. To further assess the importance of cFLIP loss upon YAP/TAZ knockdown on sensitization to ER stress, we ectopically expressed cFLIP_L_ in A549 cells and then analyzed their response to thapsigargin upon silencing YAP/TAZ expression with siRNAs. Ectopic cFLIP_L_ expression significantly inhibited YAP/TAZ knockdown-mediated sensitization to thapsigargin-induced activation of caspase-8 (Fig. 6E) and apoptosis (Fig. 6F).

Although chronic ER stress may result in YAP/TAZ exiting from the nucleus into the cytoplasm [24], our findings suggest that control of both intracellular TRAIL-R2/DR5 clustering and cFLIP expression by the YAP/TAZ-TEAD signaling module could represent a key event in the adaptive response of tumor cells to various types of inherent stress, that would otherwise ultimately lead to endoplasmic reticulum stress-induced apoptosis.

### Involvement of the YAP/TAZ-TEAD signaling pathway in the regulation of pro-inflammatory IL-8 expression in cells undergoing ER stress

**Fig. 7:**
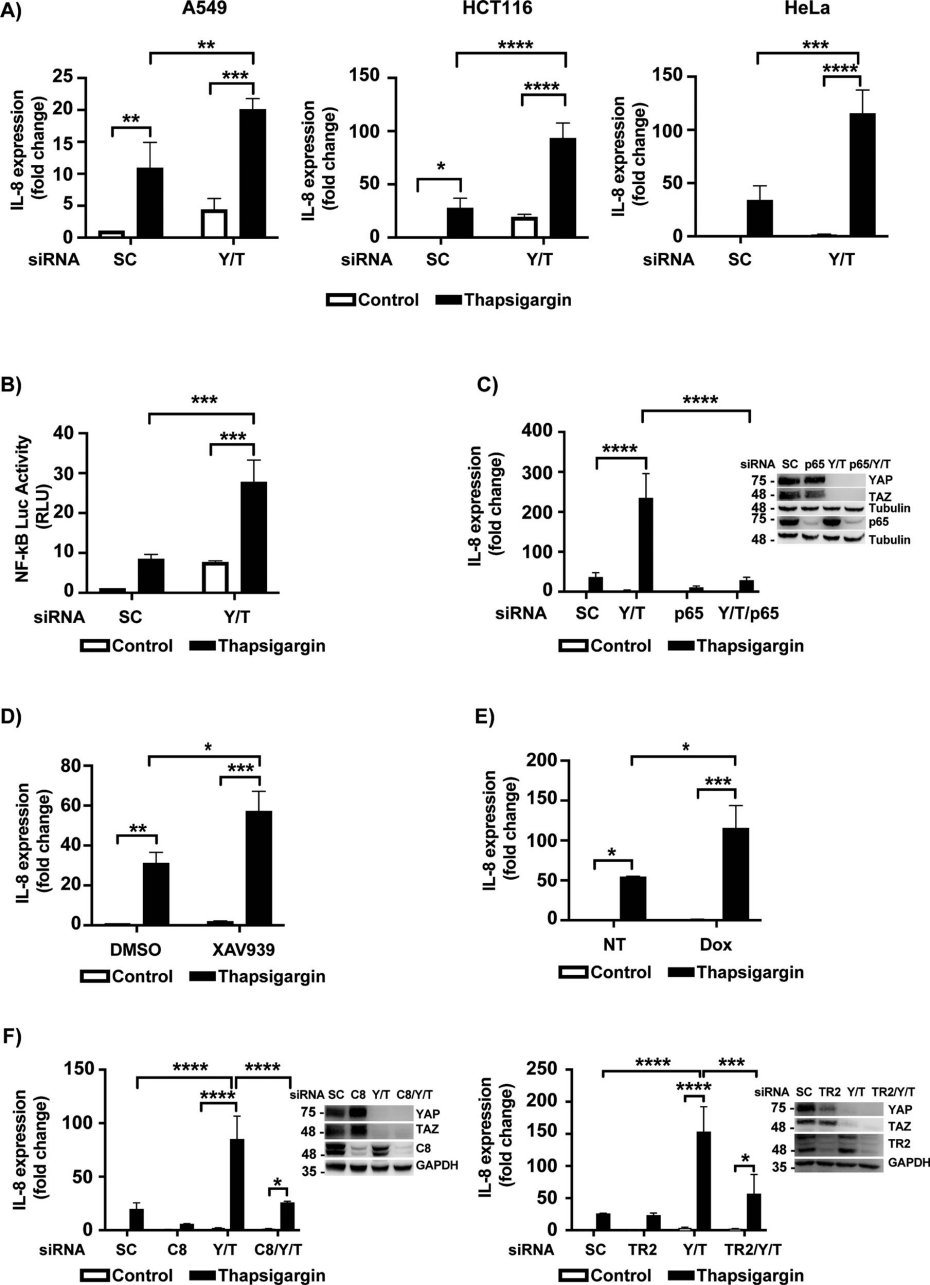
Inhibition of YAP/TAZ-TEAD signaling promotes ER-stress-induced inflammation. **A** Cells were transfected with siRNA against both YAP and TAZ (Y/T) as described in Material and Methods. 30 h post-transfection, cells were treated with thapsigargin (A549 and HeLa 200 nM, HCT116 100 nM) and QVD (20 µM) during 24 h. Interleukin 8 (IL8) mRNA levels were analyzed by qPCR. **B** HeLa cells were transfected with siRNA Y/T and, at the same time, with the NF-κB Luc reporter plasmid. Thirty hours post-transfection cells were treated with thapsigargin 24 h and NF-κB luciferase activity was measured. **C** HeLa cells were transfected with the indicated siRNAs. Thirty hours post-transfection cells were treated with thapsigargin for 24 h and IL8 mRNA was assessed by qPCR. **D** HeLa cells were cultured in the presence or absence of XAV939 5 μM during 24 h. Then thapsigargin was added during 24 h and IL8 mRNA analyzed. **E** HeLa TEADi cells were treated with doxycycline during 24 h before thapsigargin treatment for 24 h. IL8 mRNA was analyzed by qPCR. **F** HeLa cells were transfected with siRNA Y/T and caspase-8 (C8) (left panel) or siRNA Y/T and TRAIL-R2 (TR2) (right panel) and treated with thapsigargin for 24 h. IL-8 mRNA was analyzed as previously indicated. Data show mean ± SD from three independent experiments (**A**, **B**, **E**) or mean ± SEM (**C**, **D**) *P < 0.05; **P < 0.01; ***P < 0.001; ****P < 0.0001. Two-way ANOVA with Tukey´s multicomparisons test.

Recent data have shown that in addition to being responsible for the induction of apoptosis in cells subjected to stress in the endoplasmic reticulum [13–15], TRAIL-R2/DR5 is an essential element in the NF-kB-dependent activation of an inflammatory response in these stress conditions [38]. However, it is unknown whether the YAP/TAZ-TEAD module plays any role in the control of the inflammatory response induced after ER stress. To address this question, we initially assessed the expression of the inflammatory cytokine IL-8 in response to ER stress in various tumor cell lines in which YAP/TAZ expression had been silenced prior to ER stress. As shown in Fig. 7A, YAP/TAZ knockdown markedly increased IL-8 mRNA expression in cells treated with thapsigargin. Moreover, silencing YAP/TAZ expression markedly enhanced ER stress-induced NF-kB activity (Fig. 7B) that was required for thapsigargin-induced IL-8 up-regulation (Fig. 7C).

**Fig. 8:**
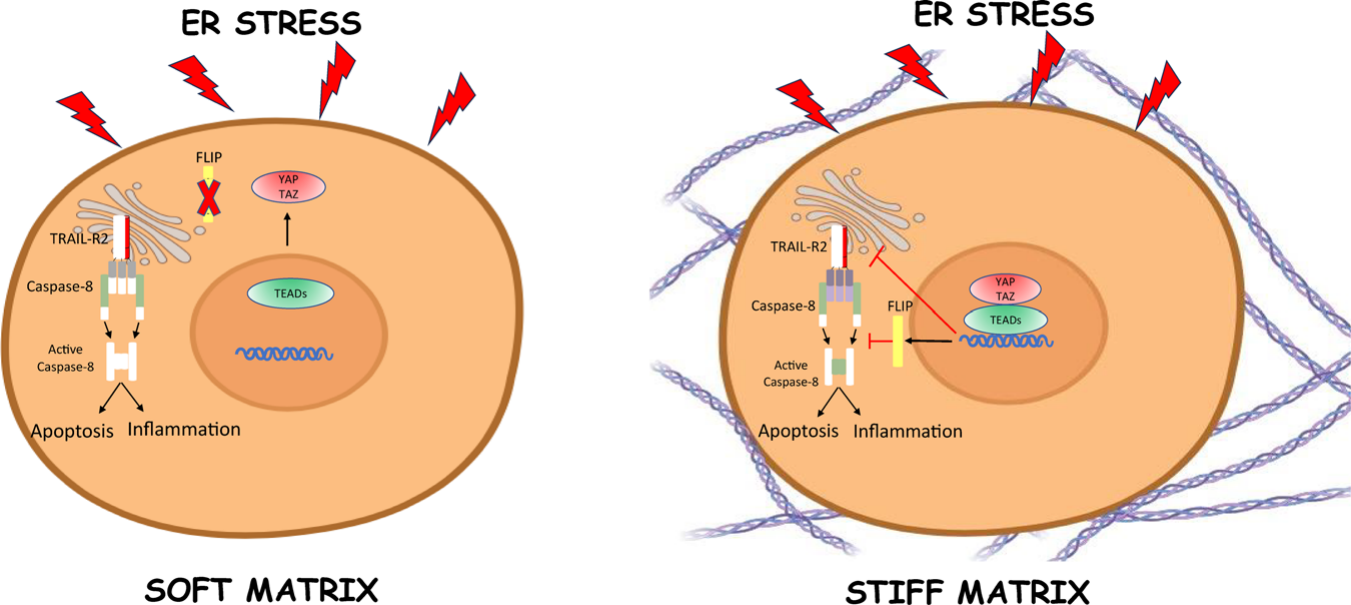
Schematic illustration of a proposed mechanism for the control by ECM stiffness of ER stress-induced apoptosis and inflammation in tumor cells.

To further assess the mechanism of the enhanced inflammatory response observed in cells depleted of YAP/TAZ, we next investigated the role of the YAP/TAZ-TEAD signaling module by analyzing the effect of the tankyrase inhibitor XAV939 on IL-8 expression levels in cells treated with thapsigargin. Inhibiting YAP/TAZ-TEAD signaling with XAV939 (Supplementary Fig. 3D) significantly enhanced thapsigargin-induced IL-8 expression in HeLa cells (Fig. 7D). Additional evidence on the role of the YAP/TAZ-TEAD module in the inflammatory response to ER stress was obtained in cells expressing the tetracycline-inducible inhibitor TEADi. Doxycycline treatment inhibited TEAD signaling (Supplementary Fig. 3D) and significantly increased thapsigargin-induced IL-8 expression (Fig. 7E). To get further insights into the molecular mechanisms underlying YAP/TAZ-TEAD-mediated control of the inflammatory response to ER stress, we assessed the role of TRAIL-R2/DR5 and caspase-8 in IL-8 expression in cells depleted of YAP/TAZ. Results shown in Fig. 7F demonstrate that silencing caspase-8 or TRAIL-R2/DR5 expression markedly inhibited IL-8 expression induced upon thapsigargin treatment in YAP/TAZ-depleted HeLa cells. Collectively, our findings reveal that in addition to restrain the activation of an apoptotic process, the YAP/TAZ-TEAD signaling module is also involved in the control of a TRAIL-R2/DR5-mediated inflammatory response in tumor cells undergoing ER stress (Fig. 8).

## Discussion

Physiological control of extracellular matrix stiffness is an essential process to maintain tissue architecture and cellular homeostasis [39, 40]. However, during tumor development, alterations in the composition and mechanical properties of the extracellular matrix occur, leading to changes in its stiffness [2–4]. These changes in ECM stiffness may lead to important changes in cytoskeletal dynamics, which in turn activate mechanotransduction signaling pathways [20, 41] that are fundamental in tumor progression [5, 6]. In addition to cell-intrinsic alterations, the uncontrolled proliferation of tumor cells in solid tumors results in the generation of different stress factors in the tumor microenvironment such as hypoxia, nutrient scarcity, and acidosis that can affect the correct folding of proteins in the ER of the tumor cells, leading to persistent ER stress [3]. Taking into account that a possible outcome of the chronic activation of the ER stress response is the induction of an apoptotic process [42], an essential question that remains unresolved is to know the mechanisms that allow tumor cells to remain viable in these conditions, thus enabling tumor progression. Our data suggest that ECM rigidity-mediated regulation of the YAP/TAZ-TEAD transcriptional module may be playing a key role in the tumor cell response to ER stress by controlling the activation of the TRAIL-R2/DR5-mediated signaling pathway, although the role of the Hippo pathway has not been addressed in our work. In addition, while YAP/TAZ might have cytoplasmic functions [43], our findings with the tankyrase inhibitor XAV939 as well as the TEAD inhibitor further suggest a role of the nuclear YAP/TAZ-TEAD module in the regulation of ER stress-induced apoptosis. Intracellular clustering of pro-apoptotic TRAIL receptor is an essential event in apoptosis activation in cells undergoing ER stress [13]. In addition, TRAIL-R2/DR5 is a key player in the inflammatory response activated in tumor cells upon ER stress [38]. Our data show for the first time that ECM rigidity is playing an essential role controlling TRAIL-R2/DR5 oligomerization and sensitivity to ER stress-induced apoptosis through a mechanism that involves the YAP/TAZ co-transcriptional activators. Our findings also reveal that the YAP/TAZ-TEAD axis functions as a relevant regulator of the pro-inflammatory response induced in tumor cells undergoing ER stress. Deciphering the mechanism responsible for TRAIL-R2/DR5 clustering in cells growing in soft ECM or depleted of YAP/TAZ is an issue that requires further investigation. In this respect, different studies have demonstrated an important role of the YAP/TAZ system in the transcriptional control of genes involved in cytoskeleton dynamics [31, 44, 45]. One possible explanation for our observation of TRAIL-R2/DR5 oligomerization at intracellular membranes of the secretory pathway in tumor cells depleted of YAP/TAZ is the inhibition of vesicle trafficking required for protein transport from the Golgi to the plasma membrane as recently reported for other receptors [31]. Alternatively, it has been reported that YAP/TAZ may have a transcriptional co-repressor function of different target genes, including TRAIL [46]. In addition, ectopic expression of TRAIL was shown to result in the intracellular retention of TRAIL receptors [47]. Therefore, reducing the nuclear levels of YAP/TAZ either by growing the cells in soft ECM or by silencing their expression would lead to TRAIL up-regulation and TRAIL-induced intracellular oligomerization of TRAIL-R2/DR5, which may trigger apoptosis signaling and inflammatory cytokine production.

Our findings reveal that besides regulating TRAIL-R2/DR5 oligomerization, ECM stiffness is also modulating a YAP/TAZ-mediated signaling mechanism to control cFLIP expression in tumor cells. Since YAP/TAZ has been reported to promote global mRNA translation by increasing mTORC1 activity [48], our data suggest that YAP/TAZ knockdown may be reducing protein synthesis which will be further inhibited upon ER stress. As both FLIP isoforms are short-lived proteins subject to ubiquitination and degradation by the proteasome [49], a reduction in the global rate of protein synthesis following ER stress could result in cFLIP loss by proteasomal degradation. In this respect, cFLIP_L_ levels play an important role in tumor cell fate upon ER stress by inhibiting early activation of TRAIL-R2/DR5-activated apoptotic pathway [37] thus granting the necessary conditions to mount an adaptive response that will restore proteostasis and support tumor progression. Along with the canonical role of cFLIP proteins controlling DISC-dependent caspase-8 activation at the plasma membrane, it has been reported that cFLIP_L_ may be also present at the ER to inhibit caspase-8-mediated cleavage of ER-localized proteins [50]. Therefore, our results underscore the importance of the increased ECM stiffness to maintain cFLIP levels and tumor cell viability in the adverse environmental conditions of the tumor microenvironment, through the activation of the YAP/TAZ-TEAD signaling module. Supporting these results, our recent data revealed that the mevalonate pathway is controlling cFLIP levels and sensitivity to TRAIL in tumor cells through the regulation of the YAP/TAZ-TEAD signaling axis [51].

Overall, our data suggest that in addition to other functions ECM stiffness could play a role in tumor cell fate in the harsh conditions of the tumor microenvironment by YAP/TAZ-mediated regulation of critical events in the activation of apoptosis and inflammation, thus promoting tumor growth and progression.

## Materials and methods

### Cell culture

HEK293-T cell line was kindly provided by Dr. A. Rodriguez (Universidad Autónoma Madrid, Spain). A549 (ATCC CCL-185), HeLa (ATCC CCL-2) and HEK293-T cells were grown in DMEM medium supplemented with 10% fetal bovine serum, 2 mM L-glutamine, penicillin (50 U/ml) and streptomycin (50μg/ml). Complete medium of A549 cells was also supplemented with glucose (4,5 g/l). HCT116 cell line was a donation of Dr. J.A. Pintor-Toro (CABIMER, Seville, Spain) were maintained in McCoy’s medium supplemented with 10% heat-inactivated fetal bovine serum, 2 mM L-glutamine, penicillin 50U/ml and streptomycin 50 μg/ml. All cell lines were grown at 37 °C in a 5% CO_2_-humidified, 95% air incubator and regularly tested for mycoplasma contamination.

### Reagents and Antibodies

Media supplements and chemical reagents for molecular biology and buffer preparation were from Merck/Sigma-Aldrich (St. Louis, MO, USA). Propidium iodide, puromycin, hygromycin, doxycycline, and DAPI were purchased from Merck/Sigma-Aldrich. Geneticin (G-418) was from Gibco (Fisher Scientific, UK). XAV939 was obtained from Selleckchem (Houston, TX. USA). Soluble human His-tagged recombinant TRAIL was produced in our laboratory as described [52]. Rat tail collagen Type I was from Merck. Sulfo-SANPAH was from Hölzel Diagnostica (Cologne, Germany). A list of the antibodies used in this work is provided in the Supplementary Materials section.

### Preparation of polyacrylamide hydrogels

Hydrogels were prepared as previously described [41]. Briefly, 25 mm diameter glass coverslips were activated with a solution of 3-(trimethoxysilyl) propyl methacrylate (Merck/Sigma-Aldrich), acetic acid and ethanol (1:1:14), washed three times with ethanol and air-dried for 10 min. To generate gels of different stiffness, different concentrations of acrylamide and bis-acrylamide were mixed (see Table 1) in a solution containing 0.5% ammoniumpersulfate and 0.05% tetramethylethylenediamine (Merck/Sigma-Aldrich). Thirty microliters of this solution was then placed on the center of coverslips and covered with 22 mm diameter glass coverslips. After gel polymerization, top coverslips were removed and the hydrogel surface was modified with the heterobifunctional crosslinker sulfo-SANPAH. Gels were then incubated with collagen overnight at 4 °C. After washing gels with PBS, cells were then trypsinized and plated on gels. As control of a more rigid surface (2 GPa), cell cultures were also performed in plastic dishes incubated overnight at 4 °C with collagen. Experiments were carried out 16 h after cell seeding.

**Table 1:** Preparation of polyacrylamide hydrogels.

	SOFT (0.5 kPa)	STIFF (144 kPa)
PBS 1×	440 µl	1975 µl
ACRILAMYDE 40%	50 µl	150 µl
BIS ACRYLAMIDE 2%	7.5 µl	150 µl
APS 10%	2.5 µl	2.5 µl
TEMED	0.25 µl	0.25 µl
FINAL VOLUME	500 µl	500 µl

### Determination of apoptosis

Cells (3 × 10^5^/well) were treated in 6-well plates as indicated in the figure legends. After treatment, hypodiploid apoptotic cells were detected by flow cytometry according to published procedures [53]. Quantitative analysis of the cell cycle and subG1 cells was carried out in a FACSCalibur cytometer using the Cell Quest software (Becton Dickinson, Mountain View, CA, USA).

### Analysis of cell viability by propidium iodide uptake

Cells (3 × 10^5^/well) were treated in 6-well plates as indicated in the figure legends. After treatment, cells are washed twice with phosphate-buffered saline (PBS)/0.1% bovine serum albumin, and incubated for 15 min on ice in the dark in the same buffer containing propidium iodide (2 μg/ml). Quantitative analysis of propidium iodide uptake was performed in a FACSCalibur cytometer using the Cell Quest Software (Becton Dickinson, Mountain View, CA, USA).

### Immunoblot analysis of proteins

Cells (3 × 10^5^) were washed with PBS and lysed in TR3 buffer (10 mM Na2HPO4, 10% Glycerol, 3% SDS). Protein content was measured with the Bradford reagent (Bio-Rad Laboratories, Hercules, CA, USA), before adding Laemmli sample buffer. Proteins were resolved on SDS-polyacrylamide mini gels and detected as described previously [53]. GAPDH was used as protein loading control. Full and uncropped western blots are uploaded as supplemental materials.

### RNA interference

siRNAs against YAP, TAZ, cFLIP, and non-targeting scrambled oligonucleotide (Supplementary Information section) were synthesized by Merck/Sigma Aldrich. HCT116 cells were transfected with siRNAs using jetPRIME (Polyplus Transfection reagent) following manufacturer instructions. After 24 h, transfection medium was replaced with regular medium, and cells were incubated for 48 h before further analysis. For siRNA transfection in other cell types, Dharmafect-1 (Dharmacon) was used as described by the manufacturer. After 6 h, transfection medium was replaced with regular medium, and cells were further incubated for the indicated times before analysis.

### Retroviral and lentiviral vectors

pQCXIH-Myc-YAP-5SA (#33093), FUW-tetO-wtYAP (#84009), FUW-tetO-EGFP (#84041), FUdeltaGW-rtTA (#19780) and pInducer20 EGFP-TEADi (#140145) vectors for stable gene expression were obtained from Addgene plasmid repository (Watertown, MA, USA). pBabe-puro vector has been described previously [53]. To perform silencing experiments, shRNAs against caspase-8 and TRAILR2 (Supplementary information section), in a pSUPER vector (OligoEngine, Seattle, WA, USA), were digested and cloned between *EcoR1 and Cla1* into a H1 promoter-driven GFP-encoding pLVTHM lentiviral vector [54]. Lentiviruses and retroviruses were produced by transfection of HEK293-T cells by the calcium phosphate method with the corresponding vectors. Lentivirus or retrovirus-containing supernatants were collected 48 h after transfection and concentrated by ultracentrifugation at 22,000 rpm for 90 min at 4 °C.

### Generation of A549 and HeLa cell lines

Cell lines were obtained after infection with lentiviruses or retroviruses and selection in culture medium containing the corresponding antibiotic: puromycin (1,5 μg/ml), hygromycin (200 μg/ml), or G-418 (1 mg/ml). Tumor cells infected with GFP-expressing lentiviruses were detected by flow cytometry.

### Immunofluorescence

Cells were grown on coverslips and fixed in 4% paraformaldehyde for 10 min at room temperature and permeabilized with 0.5% Triton X-100. Cells were then incubated with primary antibodies for 1 h at room temperature, washed with 0.1%PBS-Tween, and incubated with the appropriate fluorescent secondary antibody for 1 h. Images were acquired with an Axio Imager 2 Zeiss microscope (Zeiss, Germany). YAP nuclear localization was assessed by calculating the ratio between YAP fluorescence in the nuclear region and the cytoplasmic region immediately adjacent. Nuclear and cytoplasmic regions were previously determined by co-staining the nucleus with DAPI. Images analysis was performed using ImageJ software.

### Determination of intracellular TRAIL-R2/DR5 aggregates

TRAIL-R2/DR5 immunofluorescence and images acquisition was performed as described. We used ImageJ software to generate a ROI delimiting the area of TRAIL-R2/DR5 aggregate and then the Integrated Density was calculated. At least 100 cells from three independent experiments were analyzed.

### Quantification of TRAIL-R2/DR5 at the Golgi

For quantifying the number of Golgi with TRAIL-R2 aggregate localized, TRAIL-R2/DR5 immunofluorescence was performed as described and an anti-GM130 antibody was used to stain the Golgi area. Images were acquired with a Leica TCS SP5 Confocal Microscope and for each condition at least 100 cells from 3 independent experiments were analyzed.

### 8xGTII–lux Luciferase assay

Luciferase assays were performed in A549 cells transfected with the established YAP/TAZ-responsive reporter 8xGTII–lux (Addgene #34615) together with Renilla plasmid to normalize for transfection efficiency. Six hours after transfection, cells were treated with doxycycline (1 μg/ml, A549-TEADi cells) or XAV939 (5 μM, A549 cells) during 24 h before collection. Cell lysates were analyzed using the Dual-Luciferase Reporter Assay System (Promega, Madison, WI, USA) according to the manufacturer’s instructions. Samples were analyzed in a Varioskan Flash microplate reader (Thermo Electron Corporation, MA, USA). Every experimental condition was performed in duplicate.

### NF-KB luciferase assay

Luciferase assays were performed in HeLa cells transfected with pSI-Check2-hRluc-NFkB-firefly (Addgene #106979) together with siRNA oligos against p65. Cell lysates were analyzed using the Dual-Luciferase Reporter Assay System (Promega, Madison, WI, USA) according to the manufacturer’s instructions. Samples were analyzed in a Varioskan Flash microplate reader (Thermo Electron Corporation, MA, USA). Every experimental condition was performed in duplicate.

### Real time-qPCR

RNA was extracted using PRImeZOL (Canvax Biotech, Córdoba, Spain) reagent, following the manufacturer’s instructions. mRNA expression was analyzed in triplicate by RT-qPCR on the ABI Prism7500 sequence detection system.

Primers and probes for real-time qPCR:

Primers for SYBR green analysis:

IL-8: Forward: 5´-CTGCGCCAACACAGAAATTATTGTA

Reverse. 5´-TTCACTGGCATCTTCACTGATTCTT

GAPDH: Forward: 5′-ATGGGGAAGGTGAAGGTCG-3′

Reverse: 5′-GGGTCATTGATGGCAACAATATC-3′

TaqMan primers and probes:

cFLIP: AIN1EV0

HPRT1: Hs01003267_m1

### Statistical analysis

All data are presented as the mean ± SD or SEM of at least three independent experiments. Statistical analysis was performed using GraphPAD Prism 10 (GraphPad Software, San Diego, CA, USA). The differences among different groups were determined by the 2way ANOVA, Tukey’s multiple comparison test. *P* < 0.05 was considered significant. *P < 0.05; **P < 0.01; ***P < 0.001; ****P < 0.0001. The statistical tests employed are indicated in the figure legends.

## Supplementary information

Supplementary Materials and Methods and Supplementary Figure Legends

Supplementary Figure 1

Supplementary Figure 2

Supplementary Figure 3

Original Data

## Data Availability

All data generated or analyzed during this study are included in the main text and the supplementary information files.
