# Supplementary material for: Regulation of ER stress-induced apoptotic and inflammatory responses via YAP/TAZ-mediated control of the TRAIL-R2/DR5 signaling pathway
Source: Cell Death Discov. 2025 Feb 4;11:42. doi: 10.1038/s41420-025-02335-w (PMC11794427; doi:10.1038/s41420-025-02335-w)
Supplement: Supplementary file 1 — Supplementary Materials and Methods and Supplementary Figure Legends [file 41420_2025_2335_MOESM1_ESM.docx]

**Supplementary Materials and Methods**

*Antibodies used in this study:*

| **Antibody** | **Source** | **Identifier** |
| --- | --- | --- |
| YAP/TAZ | Santa Cruz (CA, USA) | SC-101199 |
| TRAIL-R2/DR5 | R&D Systems (Minneapolis, USA) | AF631 |
| Caspase-8 (1C12) | Cell Signaling (CA,USA) | #9746 |
| GAPDH | Santa Cruz (CA, USA) | SC-47724 |
| ATF4 | Santa Cruz (CA, USA)  Cell Signaling | SC-200  #11815 |
| CHOP | Cell Signaling | #5554S |
| p65 | Santa Cruz (CA, USA) | SC-8008 |
| GM130 | Donated by Dr. Rosa M. Ríos (CABIMER, Spain) and generated by Dr.Y Misumi (Fukuoka University, Japan) |  |
| cFLIP (7F10) | Enzo Life Science, (NY,USA) | ALX-804-961 |
| HRP goat anti-mouse antibody | DAKO (Cambridge, UK) | #P0447 |
| HRP goat anti-rabbit antibody | DAKO (Cambridge, UK) | #P0448 |
| HRP rabbit anti-goat antibody | DAKO (Cambridge, UK) | #P0449 |
| Donkey antiRabbit D-Ligth Alexa 488 | Jackson Immuno Research | #711-545-152 |
| Donkey-antiGoat Cy3 | Jackson Immuno Research | *#705-165-147* |
| Alexa Fluor 647-conjugated donkey anti-mouse | Jackson Immuno Research | #715-605-150 |

*The individual siRNAs sequences were:*

| YAP#1:  YAP#4:  TAZ#1:  TAZ#2:  FLIP:  p65:  Non-targeting control siRNA (SC) | 5’-GACAUCUUCUGGUCAGAGAdTdT-3’  5´-GGUGAUACUAUCAACCAAAdTdT-3´  5´-ACGUUGACUUAGGAACUUUdTdT-3´  5´-AGGUACUUCCUCAAUCACATdT-3´  5′-GGGACCUUCUGGAUAUUUUdTdT-3′  5' GAUUGAGGAGAAACGUAAA[dT][dT]  5’-CUUUGGGUGAUCUACGUUAdTdT-3’ |
| --- | --- |

*The YAP/TAZ pairs of siRNAs used in the different experiments were:*

siYAP/TAZ#1: siYAP#1 + siTAZ#1

siYAP/TAZ#2: siYAP#4 + siTAZ#2

*shRNAs sequences:*

| TRAIL-R2: | 5’-GATCCCC**GACCCTTGTGCTCGTTGTC**TTCAAGAGA  **GACAACGAGCACAAGGGTCT**TTTTTA-3’ |
| --- | --- |
| Caspase-8: | 5’-GATCCCC**GGAGCTGCTCTTCCGAATT**TTCAAGAG  A**AATTCGGAAGAGCAGCTCC**TTTTTA-3’ |
| Scrambled (SC): | 5’-GATCCCC**CTTTGGGTGATCTACGTTA**TTCAAGAGA  **TAACGTAGATCACCCAAAG**TTTTTA-3’ |

**Supplementary Figure Legends**

**Figure S1.**

**A)** A549 cells were plated on plastic or collagen-coated polyacrylamide gels with different rigidity as described in Material and Methods and treated with tunicamycin 1µg/ml during 72h. Cell death was analyzed by quantification of subG1 population. **B)** HeLa cells were transfected with siRNA against YAP or TAZ or both during 30h. Then, cells were treated for 24h with thapsigargin (200nM). Cell death was analyzed by quantification of subG1 population and protein expression was analyzed by western blotting. **C)** HCT116 cells were transfected as in B, treated with thapsigargin 100nM during 24h, and analyzed as in B. **D)** HeLa EGFP or HeLa wtYAP cells were transfected with siRNA against both YAP and TAZ. After 6h, cells were treated with doxycycline 1µg/ml. 30h post-transfection, thapsigargin 200nM was added during 24h. Cell death was measured by quantification of subG1 population and protein expression was analyzed by western blotting. Data represent mean ± SD of at least three independent experiments. ****P*< 0,001; *****P*< 0,0001, Two-way ANOVA with Tukey´s multicomparisons test.

**Figure S2.**

**A)** HeLa cells were transfected with siRNA against Caspase-8, YAP and TAZ, or all three at the same time. 30h post-transfection, thapsigargin 200nM was added. After 24h, cell death was measured in the cytometer by quantification of subG1 population, and protein expression was analyzed by western blotting. **B)** A549 cells were transfected with two different siRNA sequences against both YAP and TAZ during 30h. Then, cells were treated with thapsigargin 200nM for 24h and cell death was assessed by quantification of subG1 population. **C)** A549 cells were transfected with two different siRNA sequences against both YAP and TAZ as in B and treated with thapsigargin for the times indicated in the figure. Protein expression was analyzed by western botting. **D)** A549 cells were transfected as in B and TRAIL-R2 mRNA analyzed by qPCR. Data show the mean ± SD from three independent experiments. *P < 0.05; **P < 0.01; ***P < 0.001; ****P < 0.0001. Two-way ANOVA with Tukey´s multicomparisons test (A-B). Unpaired T test (D).

**Figure S3.**

**A)** Representative immunofluorescence images of TRAIL-R2/DR5 (TR2) expression in A549 cells transfected with siRNA against YAP/TAZ (Y/T) and TRAIL-R2 (TR2) during 48h. **B)** Representative immunofluorescence images of TRAIL-R2/DR5 (TR2) and YAP/TAZ (Y/T) from A549 cells transfected with Y/T siRNAs or SC oligonucleotide and treated with thapsigargin (200nM) and QVD (20μM) during 24h. White arrows indicate TRAIL-R2/DR5 aggregates (scale bars 10μm). Graph shows TRAIL-R2/DR5 aggregates integrated density. Data show the mean ± SD of at least three independent experiments. *P< 0.05; **P < 0.01; ***P < 0.001; ****P < 0.0001. Unpaired Ttest. **C)** A549 cells were transfected with YAP/TAZ siRNA or a scrambled oligonucleotide as in B and treated with tunicamycin (1µg/ml) for 24h. Protein expression was analyzed by western botting. **D)** HeLa cells (left panel) were cultured in the presence or absence of XAV939 5μM during 24h and transcriptional activity of TEAD was measured by luciferase assay with a reporter containing tandem TEAD-binding (8XGTII–lux). HeLa iTEAD cells (right panel) were treated with doxycycline 1µg/ml during 24h and transcriptional activity of TEAD was determined. Data show Mean ± SD from three independent experiments. *P < 0.05; **P < 0.01; ***P < 0.001; ****P < 0.0001. Unpaired T test.
