# Supplementary figures and images for: Regulation of ER stress-induced apoptotic and inflammatory responses via YAP/TAZ-mediated control of the TRAIL-R2/DR5 signaling pathway
Source: Cell Death Discov. 2025 Feb 4;11:42. doi: 10.1038/s41420-025-02335-w (PMC11794427; doi:10.1038/s41420-025-02335-w)

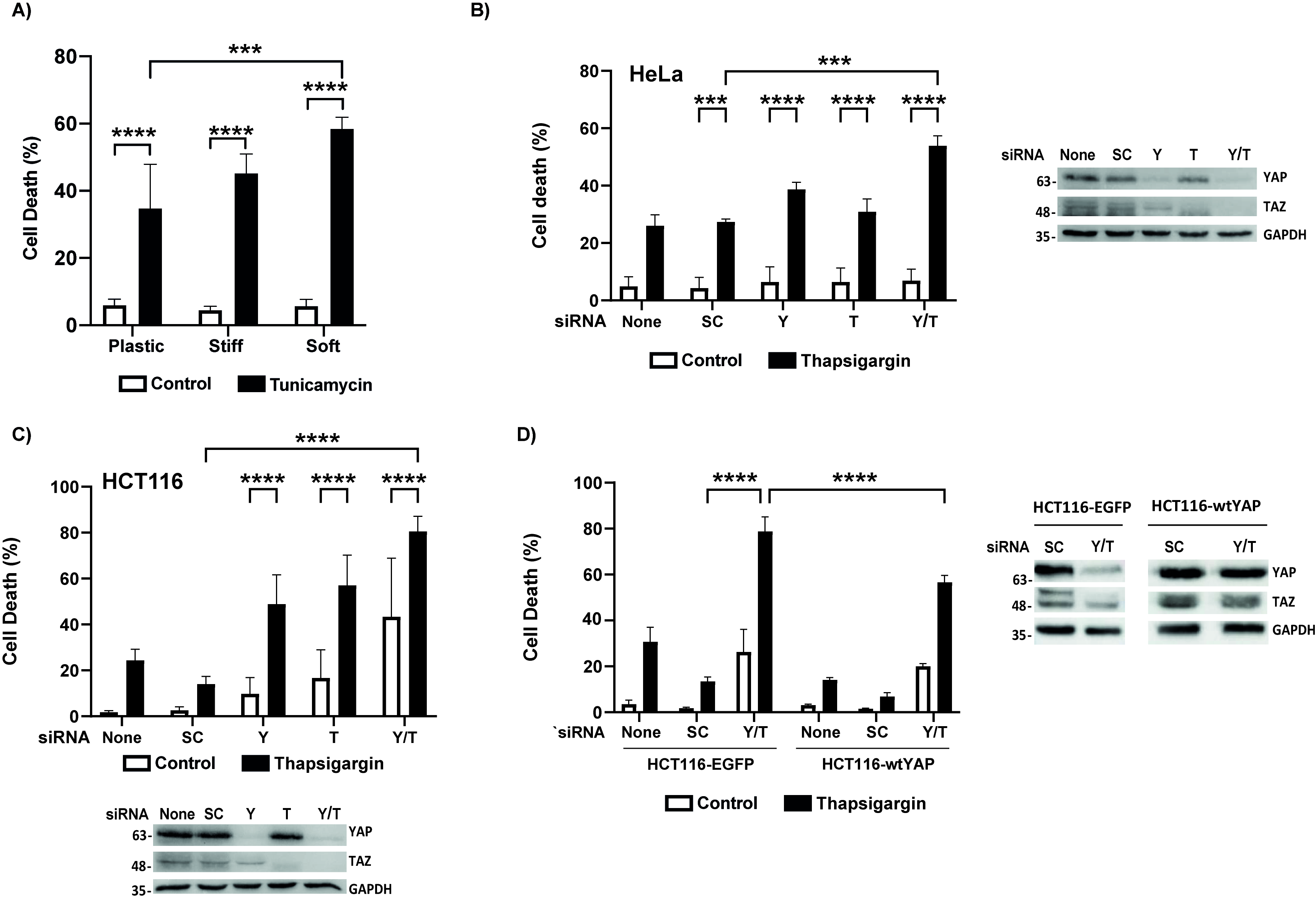

Supplement: Supplementary file 2 — Supplementary Figure 1 [file 41420_2025_2335_MOESM2_ESM.tif]

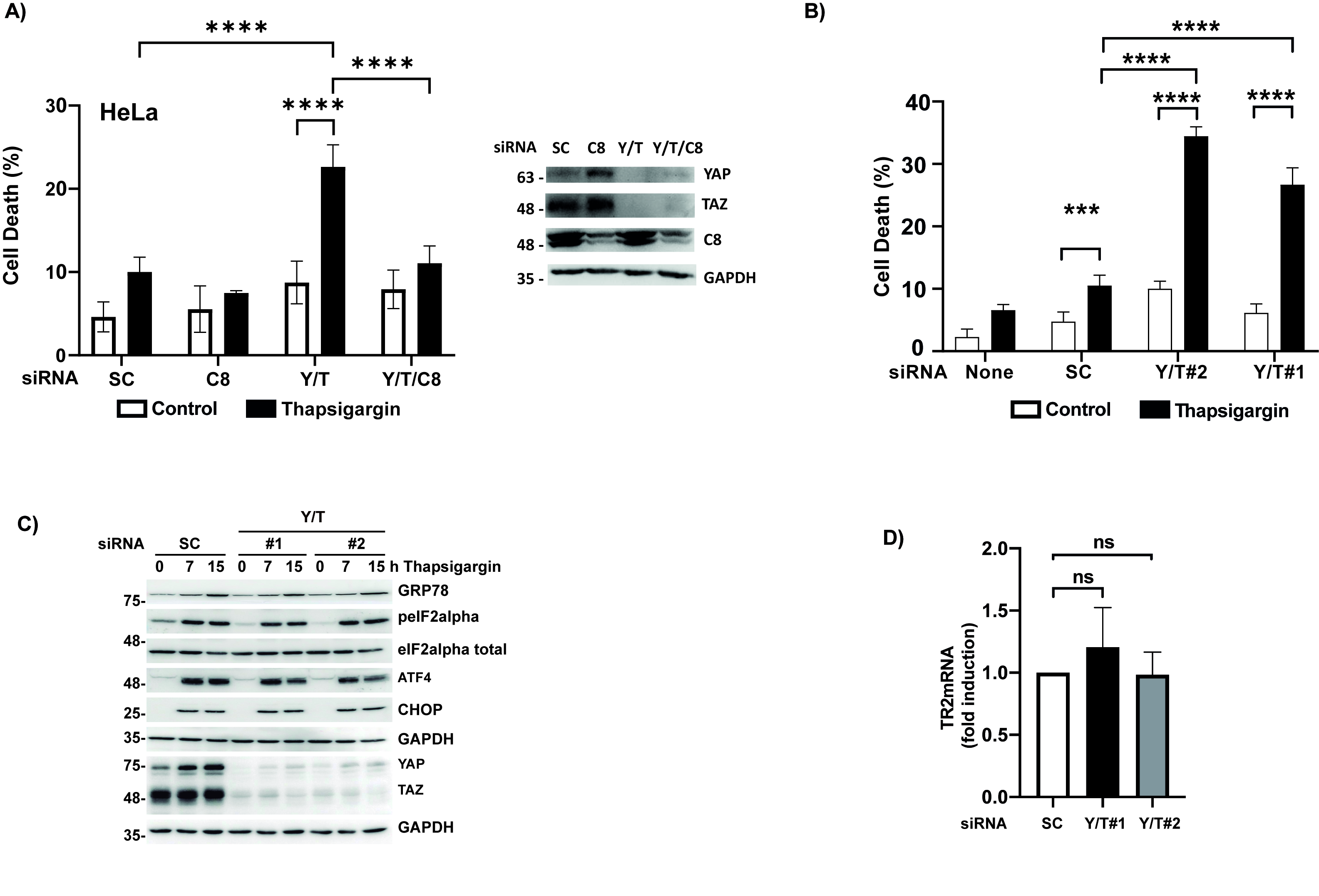

Supplement: Supplementary file 3 — Supplementary Figure 2 [file 41420_2025_2335_MOESM3_ESM.tif]

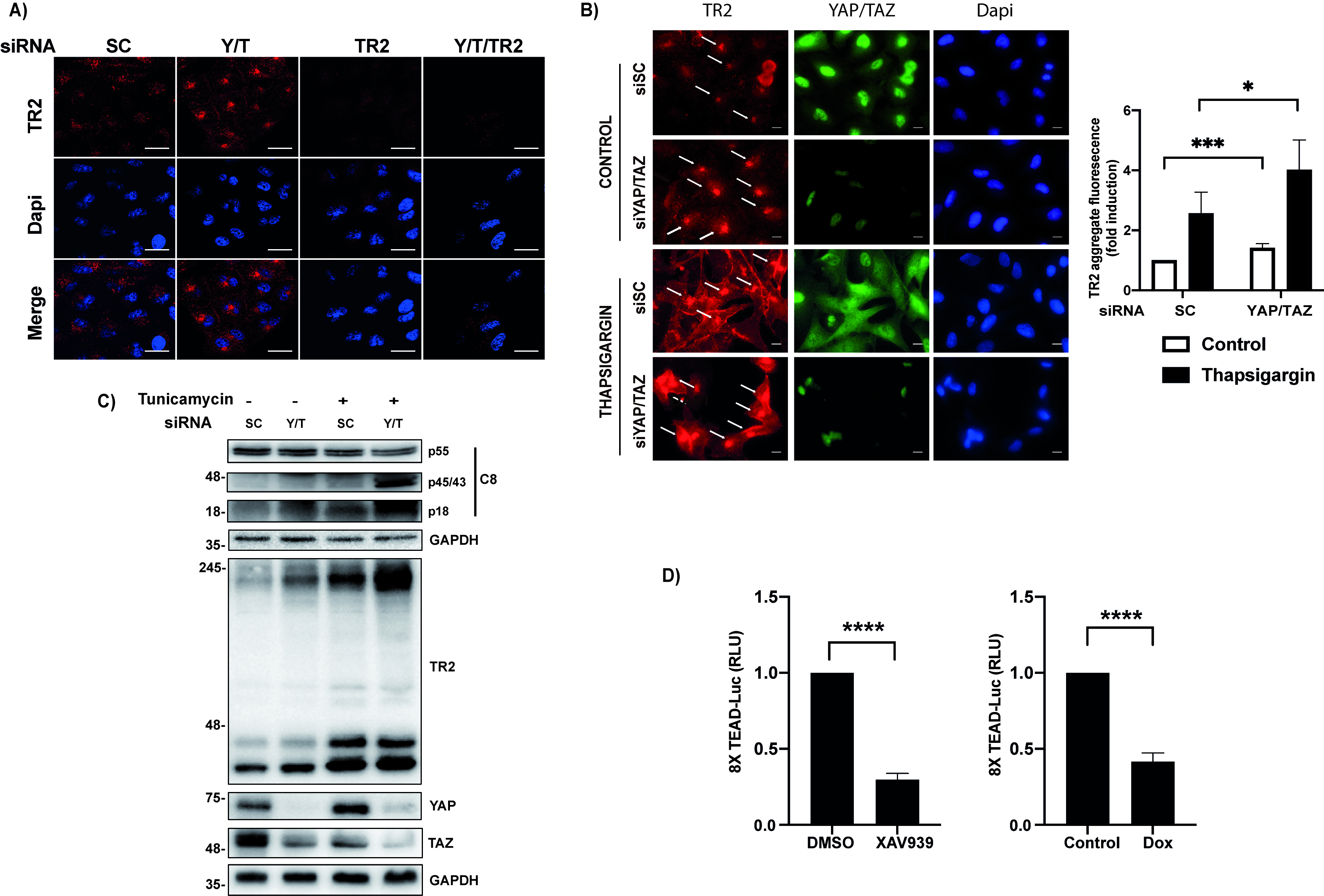

Supplement: Supplementary file 4 — Supplementary Figure 3 [file 41420_2025_2335_MOESM4_ESM.tif]
