# Supplementary material for: Regulation of ER stress-induced apoptotic and inflammatory responses via YAP/TAZ-mediated control of the TRAIL-R2/DR5 signaling pathway
Source: Cell Death Discov. 2025 Feb 4;11:42. doi: 10.1038/s41420-025-02335-w (PMC11794427; doi:10.1038/s41420-025-02335-w)

Figure 1B

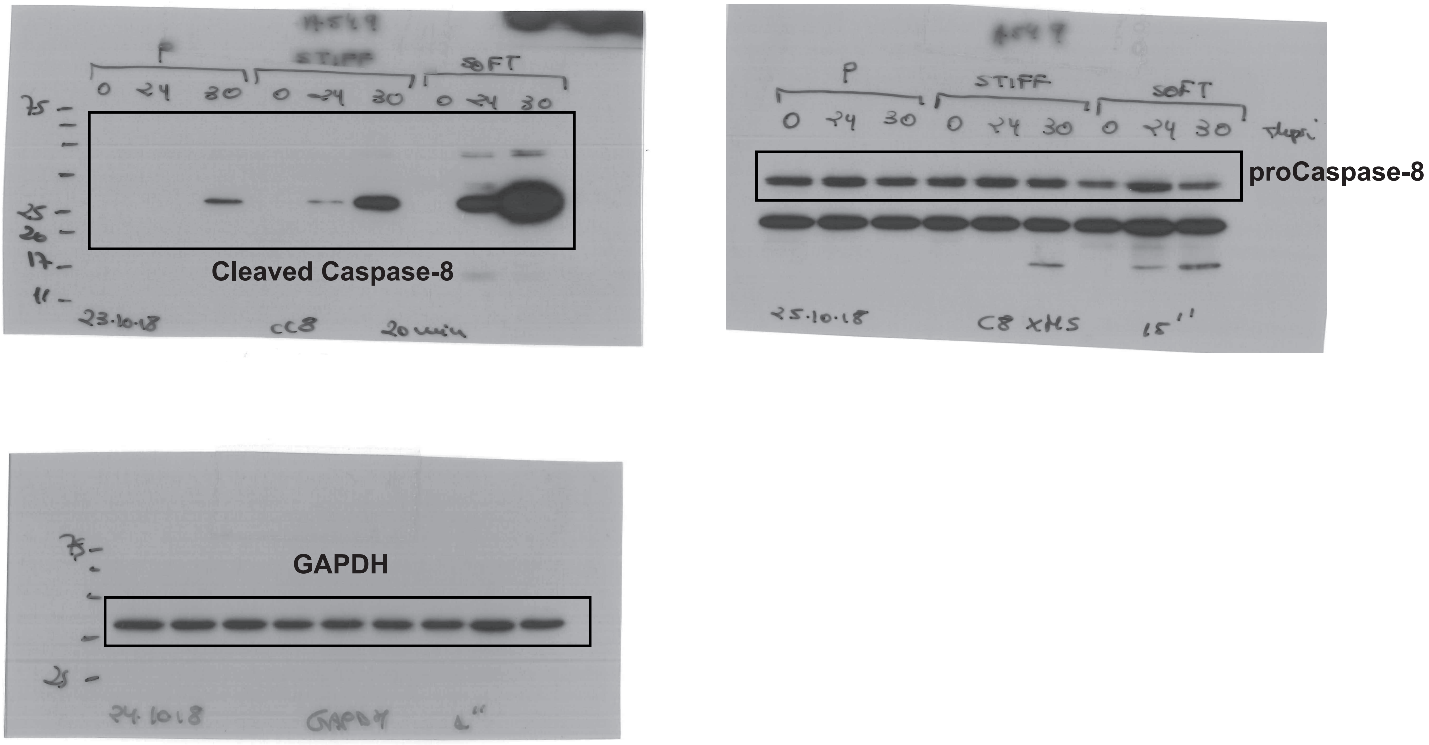

Figure 1C

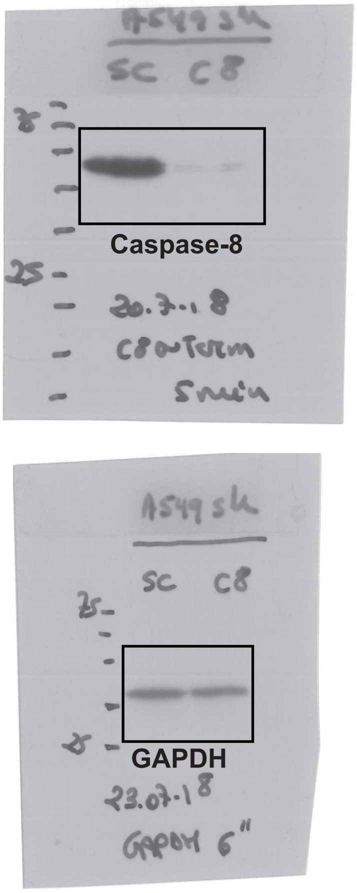

Figure 1D

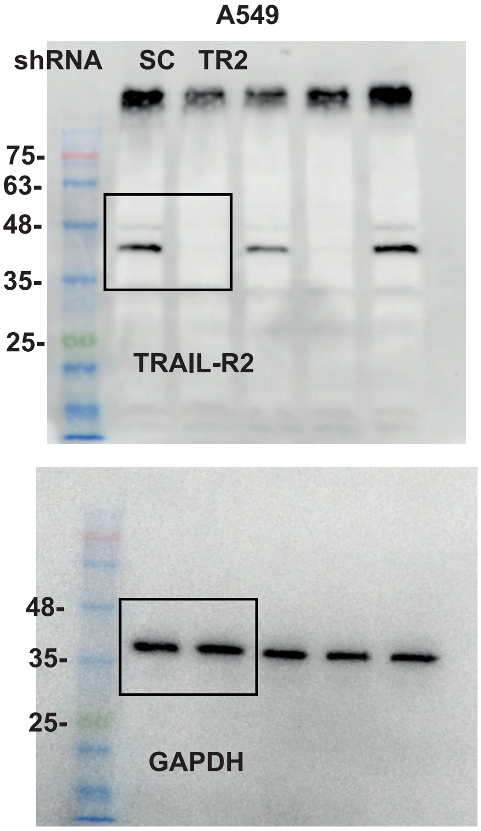

Figure 2B

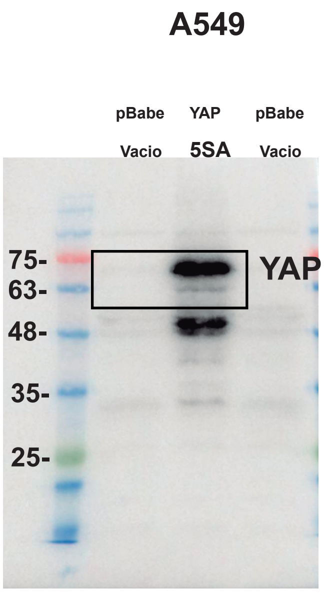
A549
pBabe Vacio
YAP 5SA
pBabe Vacio
75-
63-
48-
35-
25-
YAP

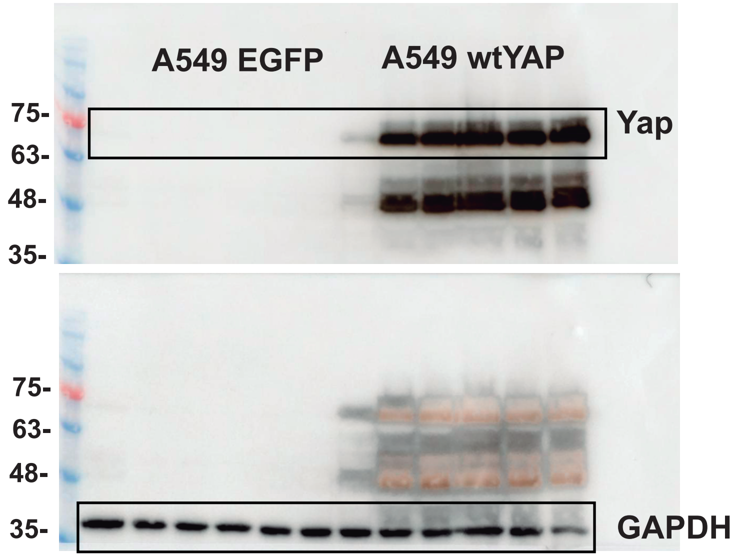
A549 EGFP
A549 wtYAP
75-
63-
48-
35-
Yap
75-
63-
48-
35-
GAPDH

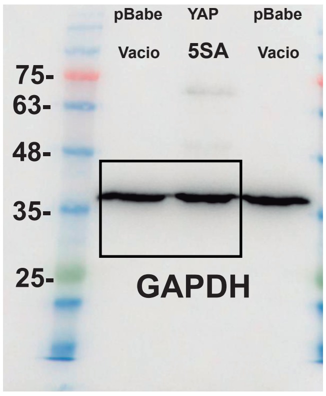
pBabe Vacio
YAP 5SA
pBabe Vacio
75-
63-
48-
35-
25-
GAPDH

Figure 3A

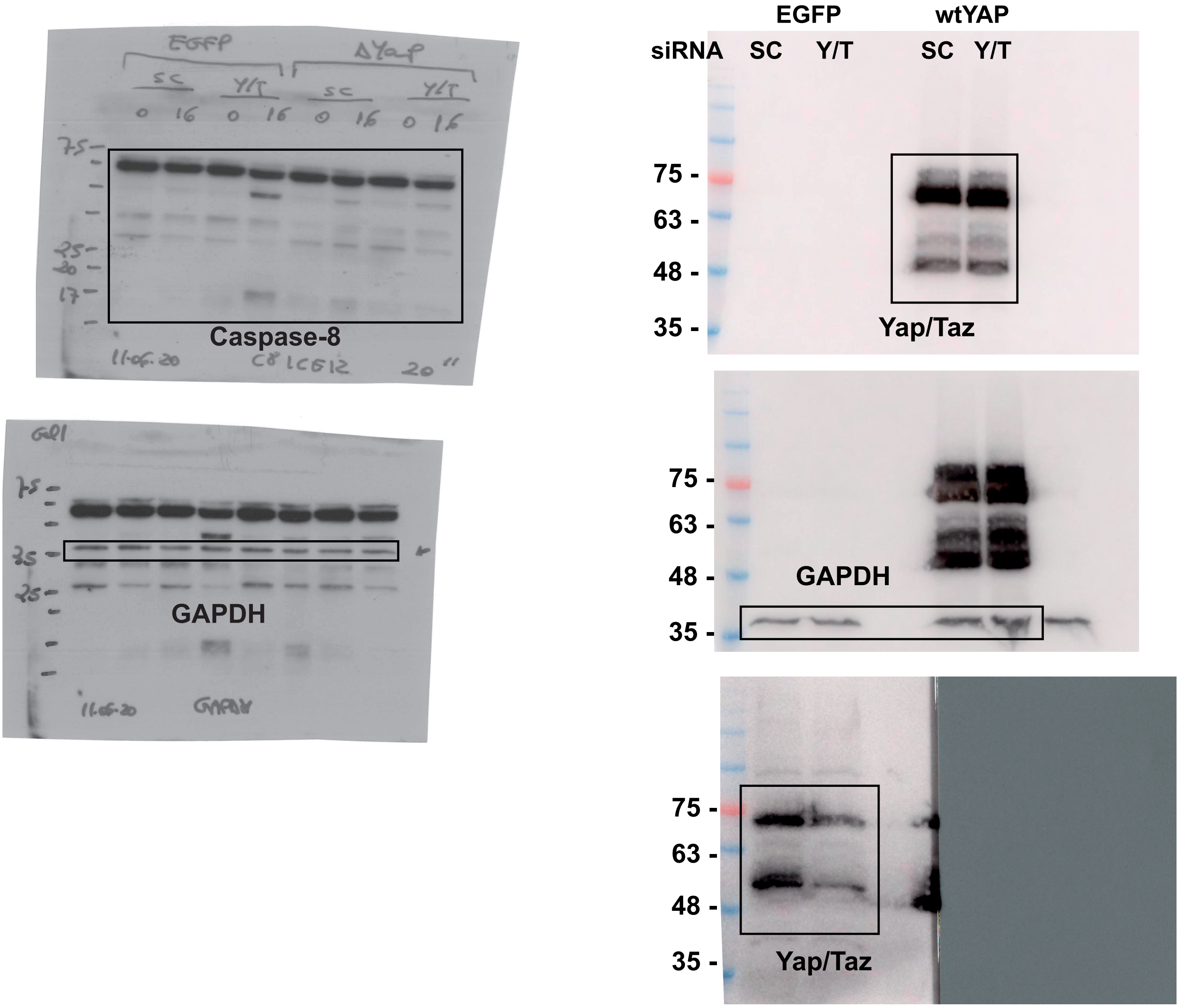
EGFP
ΔYap
Caspase-8
GAPDH
EGFP
wtYAP
siRNA
SC
Y/T
SC
Y/T
75 -
63 -
48 -
35 -
Yap/Taz
GAPDH
Yap/Taz

Figure 3D

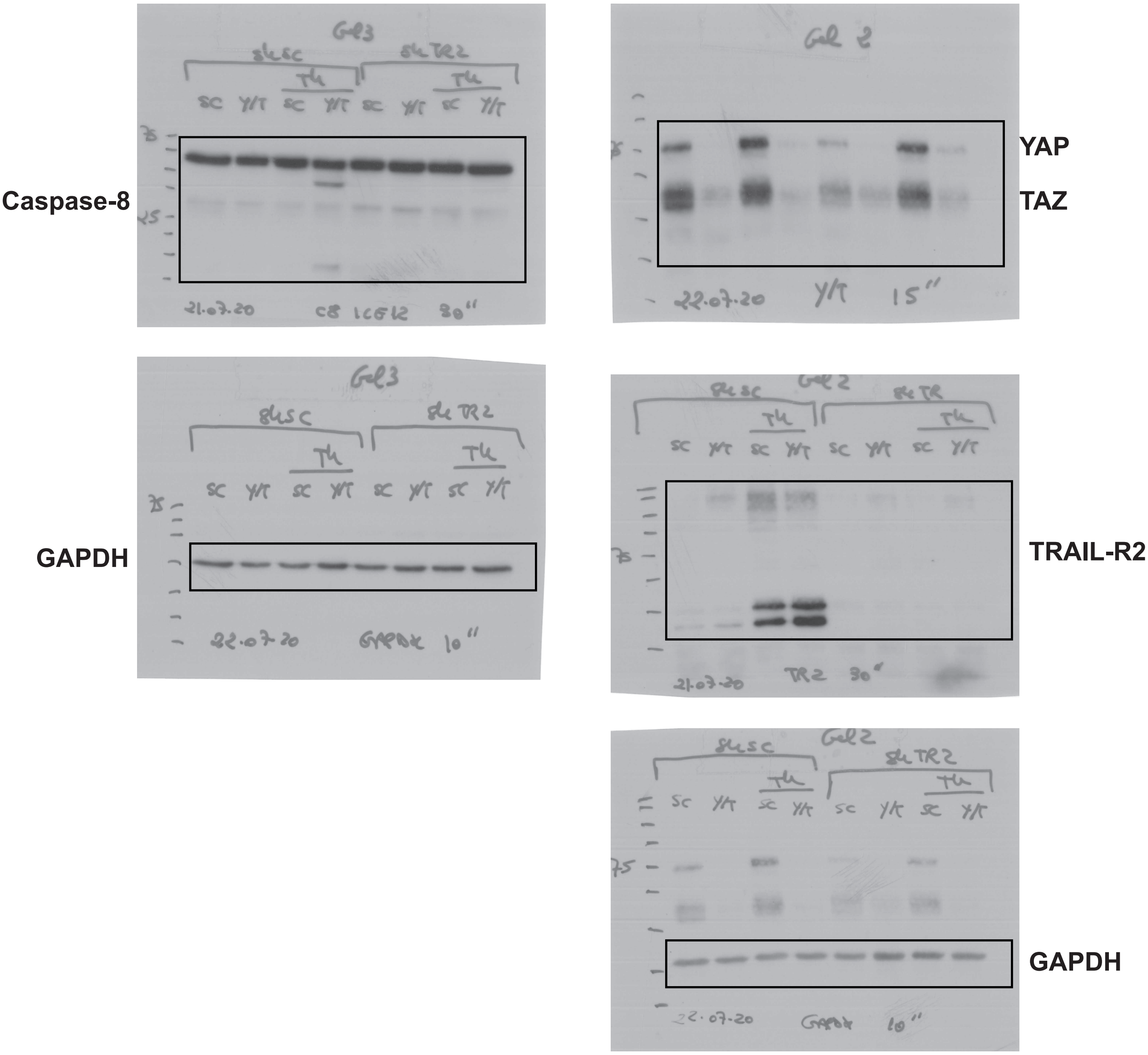
Caspase-8
GAPDH
YAP
TAZ
TRAIL-R2
GAPDH
Gel3
Gel 2
21.07.20
22.07.20
GAPDH 10''
Y/T 15''

Figure 4B

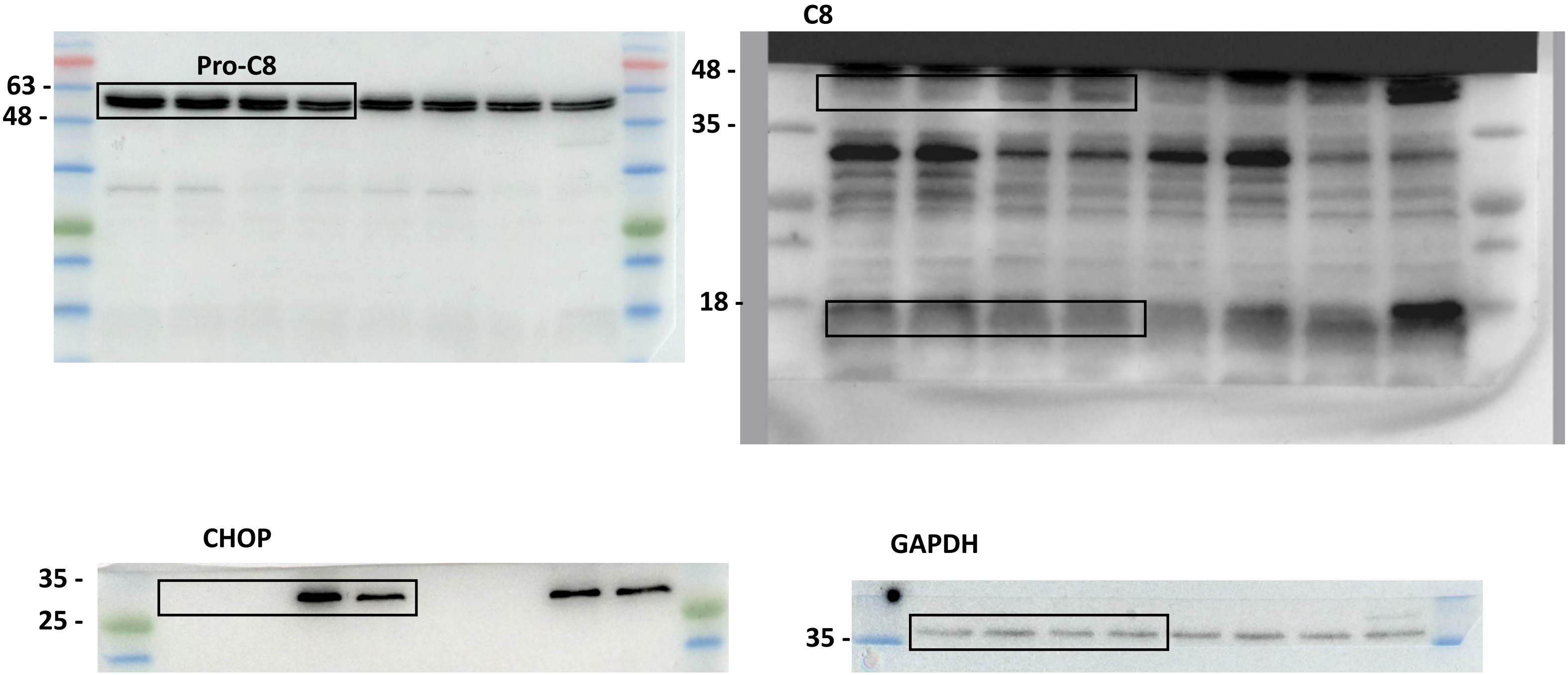

Figure 5A

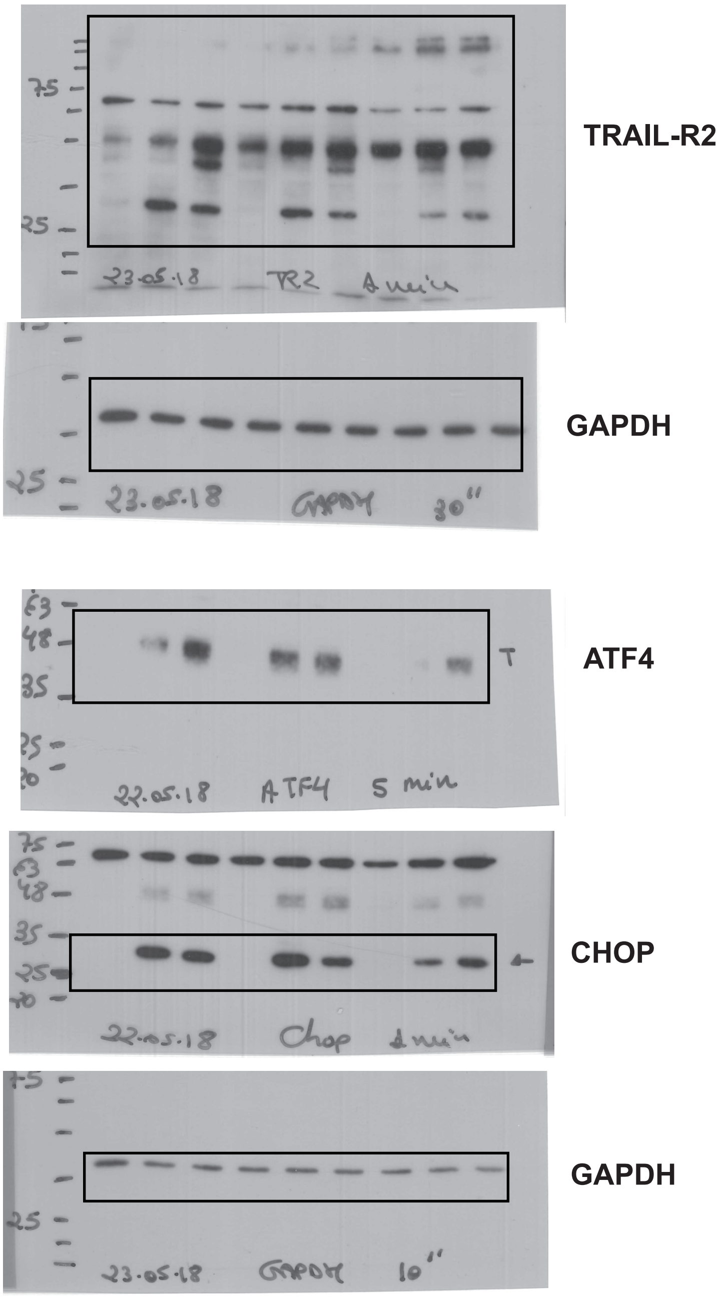

Figure 5B

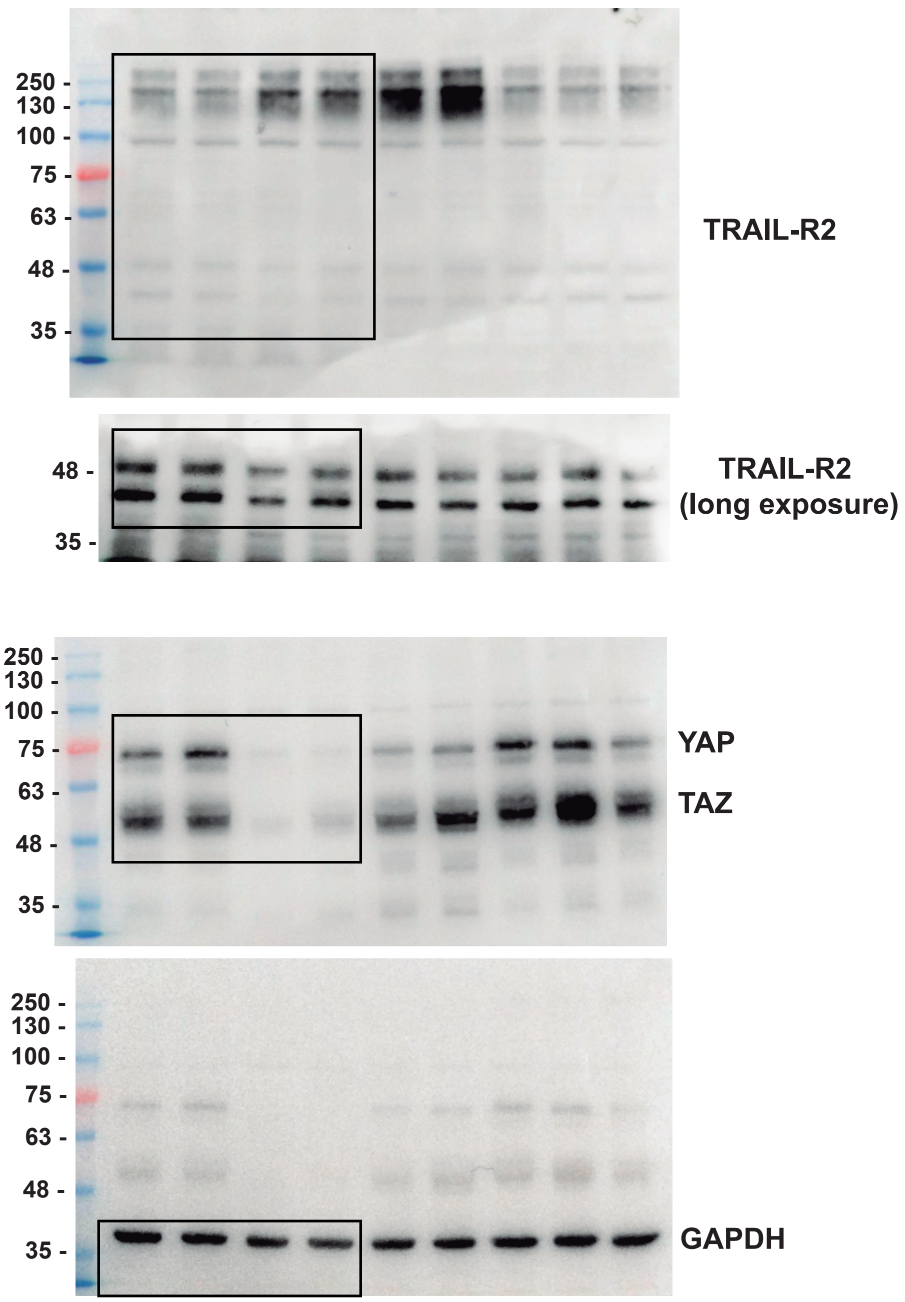
250 -
130 -
100 -
75 -
63 -
48 -
35 -
TRAIL-R2
48 -
35 -
TRAIL-R2
(long exposure)
250 -
130 -
100 -
75 -
63 -
48 -
35 -
YAP
TAZ
250 -
130 -
100 -
75 -
63 -
48 -
35 -
GAPDH

Figure 6A

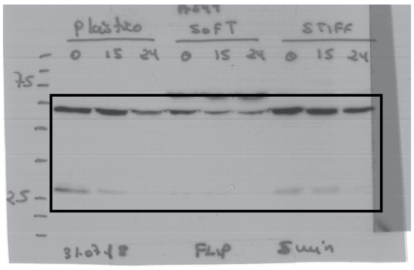

FLIP

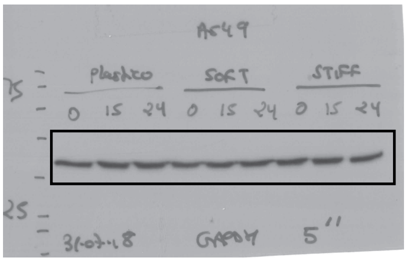

GAPDH

Figure 6B

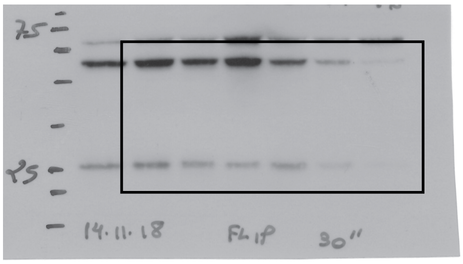

FLIP

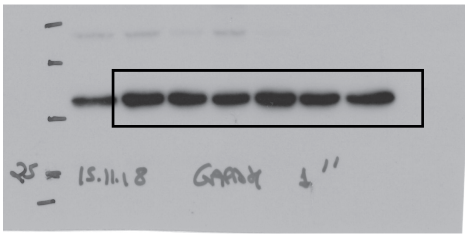

GAPDH

Figure 6E

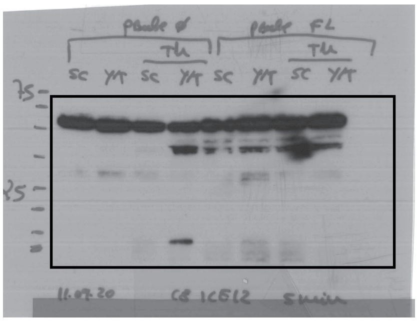

Caspase-8

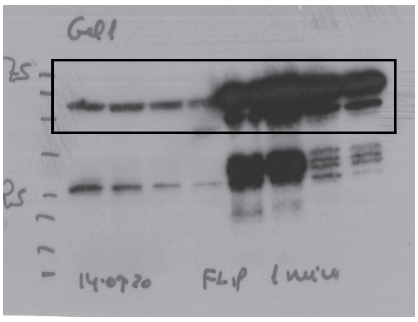

FLIP

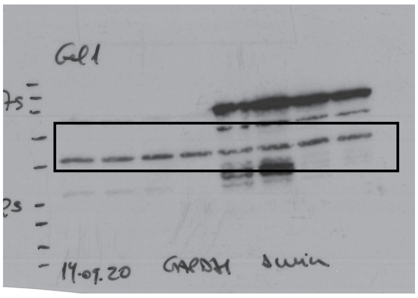

GAPDH

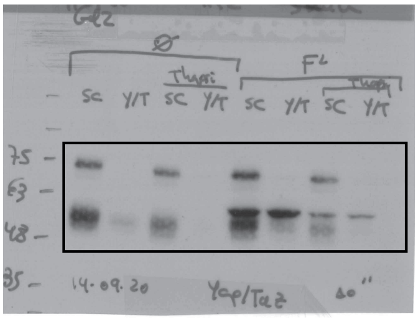

YAP/TAZ

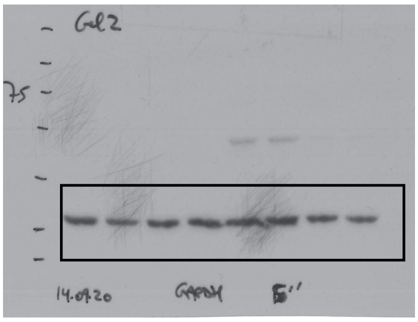

GAPDH

Figure 7B

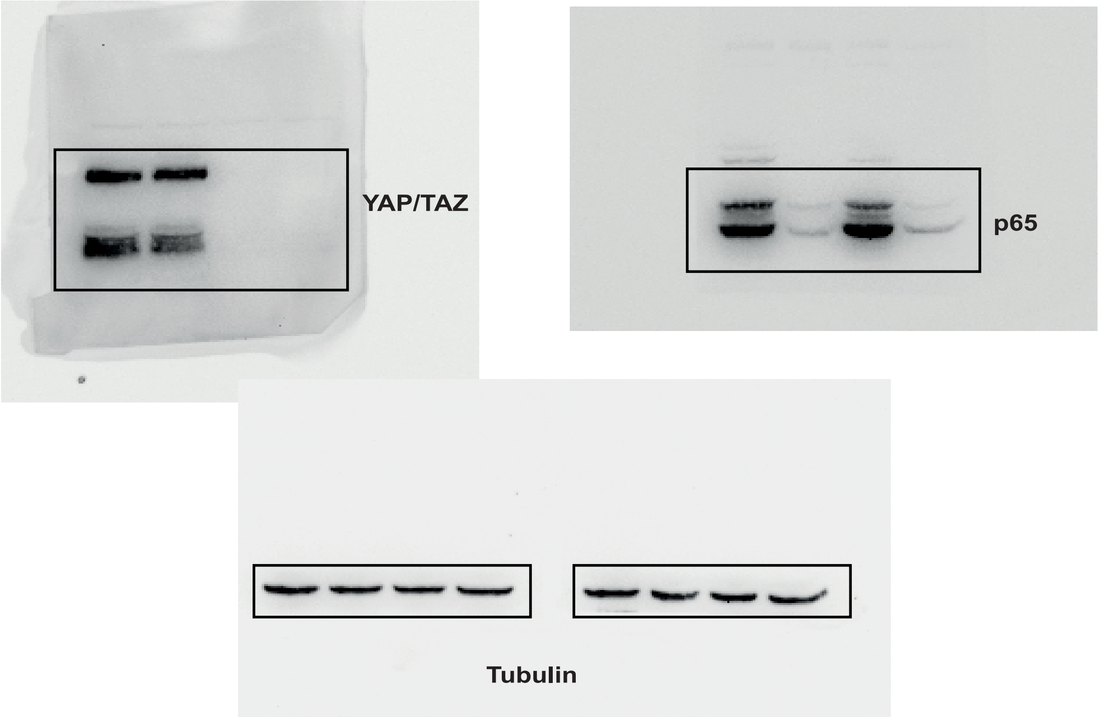

Figure 7E

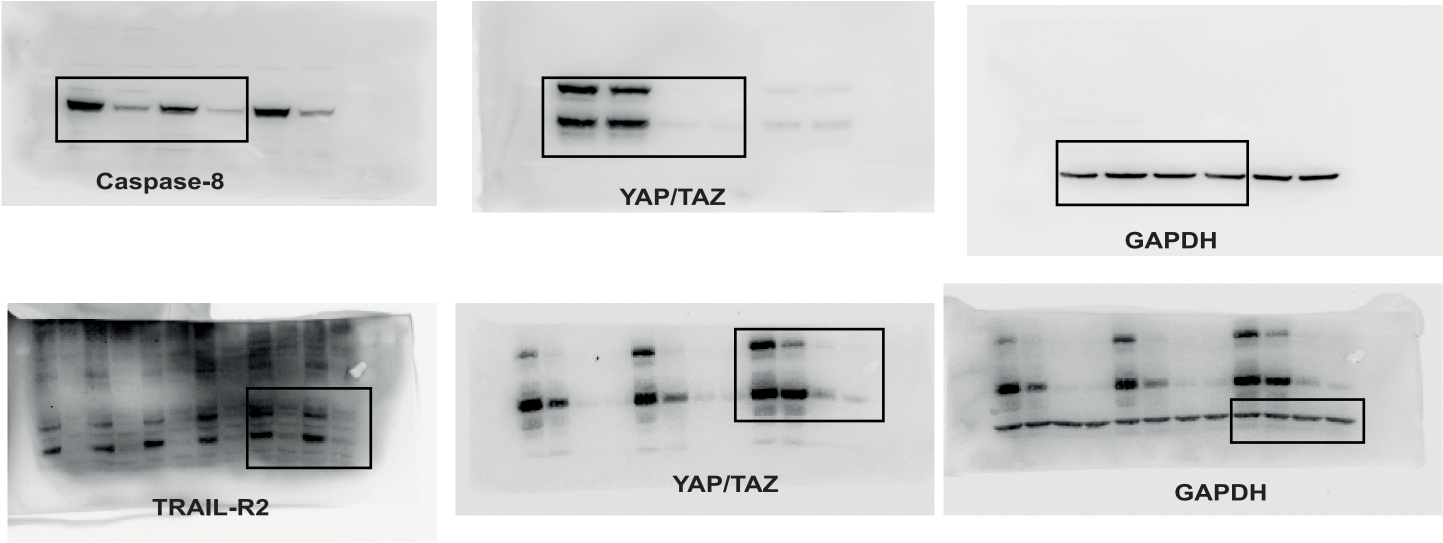

Sup. Figure 1B

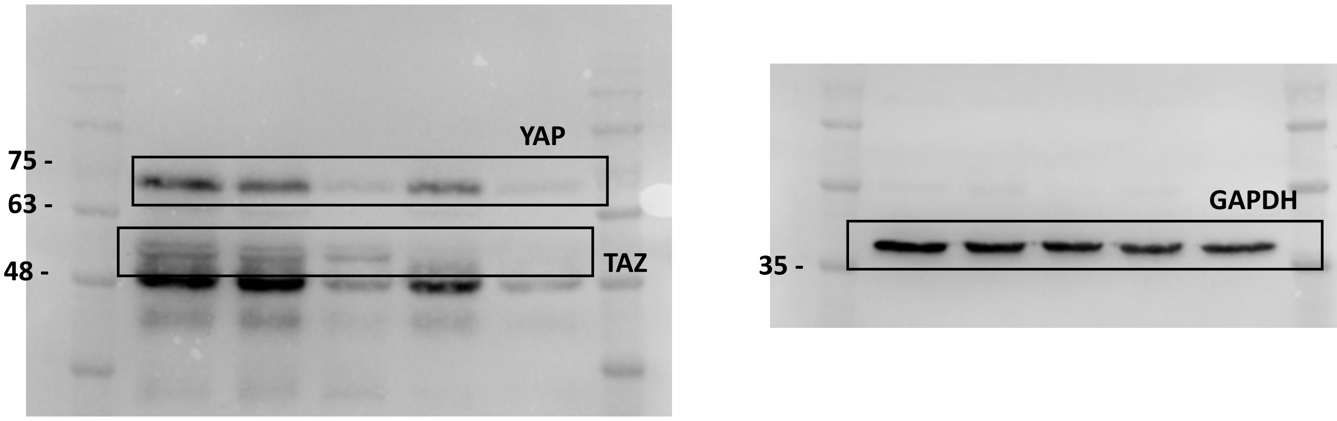

Sup. Figure 1C

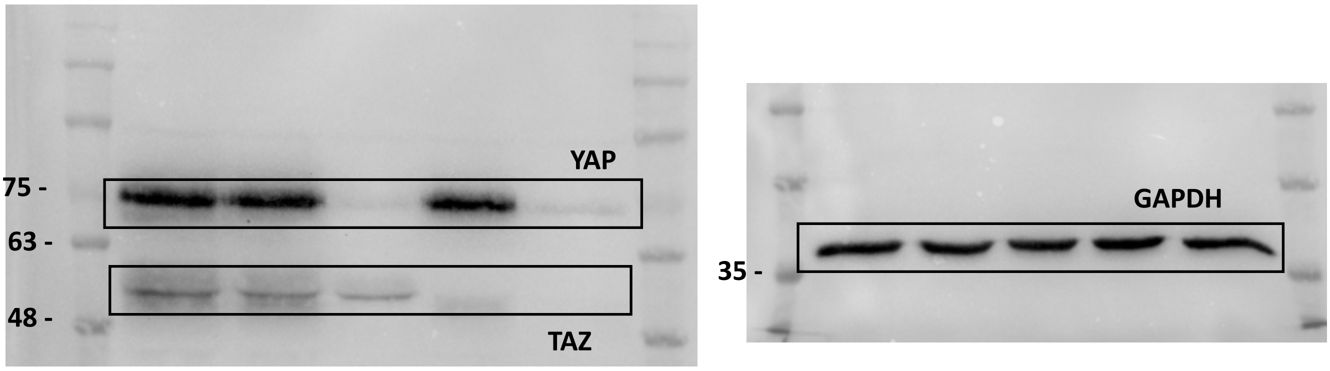

Sup. Figure 1D

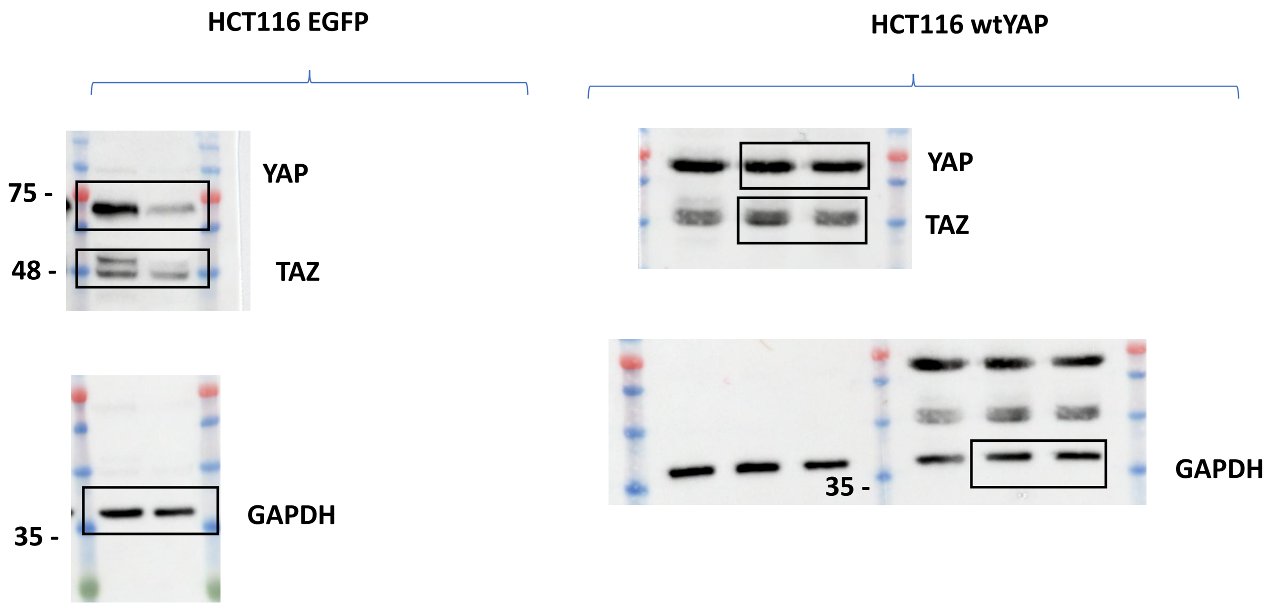

Sup. Figure 2A

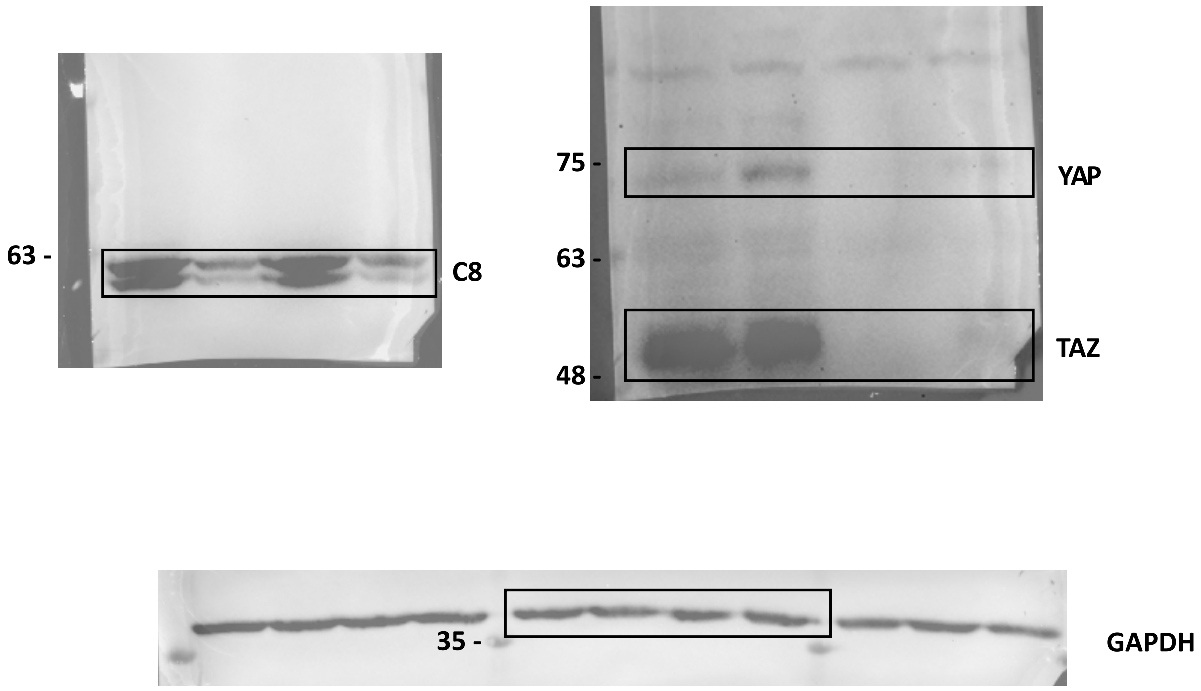

Sup. Figure 2C

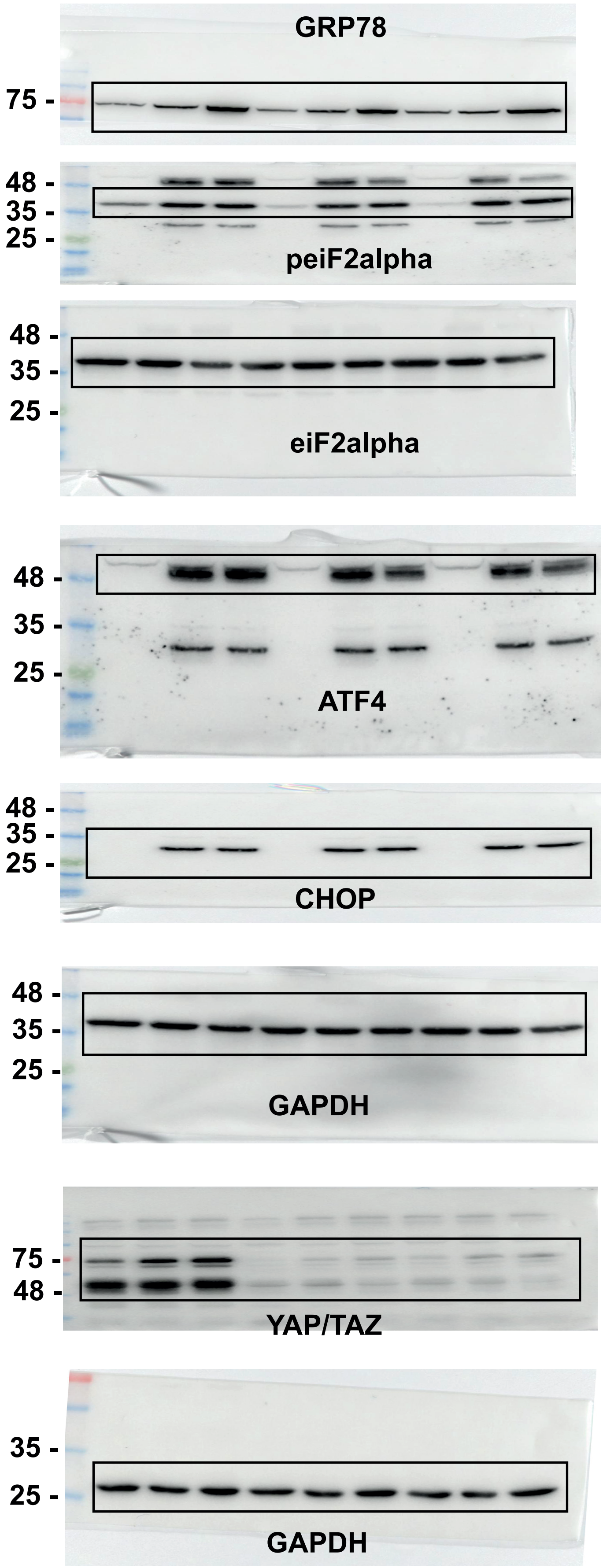

Supp. Figure 3C

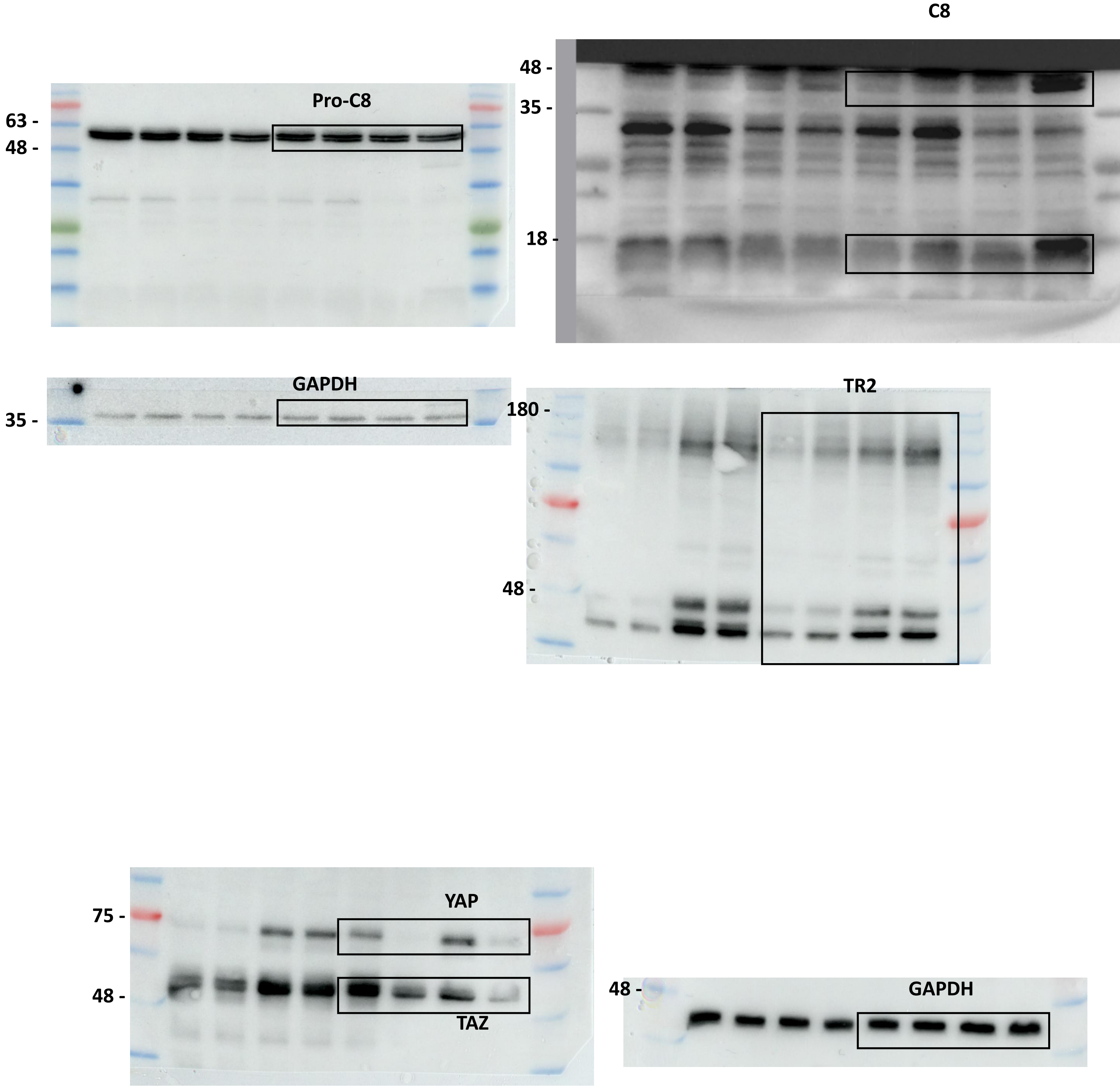

Supplement: Supplementary file 5 — Original Data [file 41420_2025_2335_MOESM5_ESM.pdf]
